# Strive or thrive: Trends in *Phytophthora capsici* gene expression in partially resistant pepper

**DOI:** 10.3389/fpls.2022.980587

**Published:** 2022-11-21

**Authors:** Gaëtan Maillot, Emmanuel Szadkowski, Anne Massire, Véronique Brunaud, Guillem Rigaill, Bernard Caromel, Joël Chadœuf, Alexandre Bachellez, Nasradin Touhami, Ingo Hein, Kurt Lamour, Sandrine Balzergue, Véronique Lefebvre

**Affiliations:** ^1^INRAE, GAFL, Montfavet, France; ^2^Université Paris-Saclay, CNRS, INRAE, Université Evry, Institute of Plant Sciences Paris-Saclay (IPS2), Gif-sur-Yvette, France; ^3^Université Paris Cité, CNRS, INRAE, Institute of Plant Sciences Paris-Saclay (IPS2), Gif-sur-Yvette, France; ^4^LaMME, Université d'Evry Val d'Essonne, INRAE, Evry, France; ^5^Division Plant Sciences at the JHI, School of Life Sciences, University of Dundee, Dundee, United Kingdom; ^6^James Hutton Institute (JHI), Dundee, United Kingdom; ^7^Department of Entomology and Plant Pathology, University of Tennessee, Knoxville, TN, United States

**Keywords:** *Capsicum annuum*, partial plant resistance, pathogen adaptation, *Phytophthora capsici*, RXLR effector, transcriptomics

## Abstract

Partial resistance in plants generally exerts a low selective pressure on pathogens, and thus ensuring their durability in agrosystems. However, little is known about the effect of partial resistance on the molecular mechanisms of pathogenicity, a knowledge that could advance plant breeding for sustainable plant health. Here we investigate the gene expression of *Phytophthora capsici* during infection of pepper (*Capsicum annuum* L.), where only partial genetic resistance is reported, using Illumina RNA-seq. Comparison of transcriptomes of *P. capsici* infecting susceptible and partially resistant peppers identified a small number of genes that redirected its own resources into lipid biosynthesis to subsist on partially resistant plants. The adapted and non-adapted isolates of *P. capsici* differed in expression of genes involved in nucleic acid synthesis and transporters. Transient ectopic expression of the RxLR effector genes CUST_2407 and CUST_16519 in pepper lines differing in resistance levels revealed specific host-isolate interactions that either triggered local necrotic lesions (hypersensitive response or HR) or elicited leave abscission (extreme resistance or ER), preventing the spread of the pathogen to healthy tissue. Although these effectors did not unequivocally explain the quantitative host resistance, our findings highlight the importance of plant genes limiting nutrient resources to select pepper cultivars with sustainable resistance to *P. capsici*.

## 1 Introduction

Plants are surrounded by countless potentially pathogenic microorganisms and yet plant disease is a rare occurrence in nature, as plants have evolved different mechanisms to prevent infection by microorganisms. Pre-formed physical and chemical barriers established by the plant impede pathogen penetration (Dixon, 2001; Hématy et al., 2009; Malinovsky et al., 2014). Pathogens that are able to bypass these barriers are confronted with a complex plant immune system triggered when the plant perceives either self-damaged plant molecules (DAMPs, Damage-Associated Molecular Patterns) or pathogen-derived molecules, such as PAMPs (Pathogen-Associated Molecular Patterns) and secreted effectors. According to the intensity of the plant response, the resistance is either qualitative when the disease progression is stopped, or quantitative when the pathogen development is reduced. Deployment of qualitative resistance at a broad scale in agrosystems applies a strong pressure on pathogens and frequently results in selection of a virulent strain that breaks down the plant’s resistance (McDonald and Linde, 2002). By contrast, quantitative resistance is generally broad spectrum and exerts a lower selective pressure, limiting the selection of virulent variants. Indeed, this type of resistance is usually more durable than major genes in agrosystems (Palloix et al., 2009; Cowger and Brown, 2019), even if adaptation of a few pathogen populations to quantitative resistance have already been reported (Delmotte et al., 2014). Understanding the molecular dialogs between plants and pathogens should enable plant breeding to achieve sustainable disease control aimed at reducing pesticides in agriculture.

In the past decades, plant-pathogen interaction studies have brought to light pathogenicity genes and qualitative resistance genes involved in gene-for-gene recognition and molecular mechanisms responsible for breakdown of resistance (Kushalappa et al., 2016). Direct or indirect molecular recognition between a plant resistance protein and a pathogen effector (avirulence factor) from a specific race or isolate triggers the resistance response. Evolved effectors (virulent factors) from adapted pathogens frequently overcome simple inherited host resistance. Gene expression studies have shed light on the transcriptomic changes occurring during compatible plant-pathogen interactions and showed that biotrophs exploit host pathways to extract nutrients from living plant cells, while necrotrophs produce toxins and cell-wall degrading enzymes (CWDE) to kill and feed on dead plant cells (Laluk and Mengiste, 2010). However molecular mechanisms during incompatible plant-pathogen interactions, particularly in the case of quantitative resistance, are much less understood.

*Phytophthora capsici* is a hemi-biotrophic oomycete plant pathogen that transitions from an early biotrophic phase to a necrotrophic phase (Fawke et al., 2015). Some *Phytophthora* effectors are secreted into the host extracellular space and interfere with apoplastic plant proteins; CWDEs breach the host cell wall, and protease inhibitors and secreted proteases counter host defenses (Kamoun, 2006; Schornack et al., 2009). Other effectors, namely members of the RxLR and Crinkler (CRN) families, are translocated into the plant cytoplasm through the oomycete haustorium membrane (Wang et al., 2017). Some apoplastic and cytoplasmic effectors have been shown to suppress plant immunity. Secreted RxLR effectors are major virulence determinants of oomycetes and avirulence forms are recognized by plant resistance proteins, resulting in the complete host immunity. Several studies reported the temporal expression of *Phytophthora* effectors during the pathogen development or host plant colonization. *P. capsici* genes, including RxLRs, CRNs, elicitins, transglutaminase elicitors, NLPs (Nep1-like proteins), CBELs (Cellulose Binding, Elicitor, and Lectin-like) and enzyme inhibitors, are differentially expressed in mycelium, zoospores and germinating cysts (Chen et al., 2013). *P. capsici* effector genes are differentially transcribed between the biotrophic and necrotrophic phases in tomato (Jupe et al., 2013). *P. infestans* genes involved in nutrient transport, conversion of energy and proteasome activities are particularly active at the mycelium stage, while pathogenicity genes are up-regulated in zoospores and germinated cysts (Ah-Fong et al., 2017). Expression of *P. infestans* transporters greatly fluctuates during the biotrophic phase in potato compared to mycelium grown on an artificial medium (Abrahamian et al., 2016). Similarly, *P. infestans* genes show temporal transcriptional regulation when infecting susceptible tomato (Zuluaga et al., 2016b). While progress has been made on how the pathogen perturbs plant processes during a compatible interaction, our goal is to better understand the effects of quantitative resistance on pathogen’s gene expression, especially the effector repertoire, and how the gene expression of the pathogen is modulated according to its adaptation to the host plant.

*P. capsici* is known to cause root, crown and fruit rot, and foliar blight in many crops including Solanaceae and Cucurbitaceae. In the present study, we report on the *in planta* transcriptome of two *P. capsici* isolates, one adapted to pepper (*Capsicum annuum* L.) and one non-adapted. We analyze how the host plant impacts on the regulation of *P. capsici* genes at early stages of the interaction, by comparing the *P. capsici* transcriptomes in a susceptible and a partially resistant pepper host. Comparison of *in planta* transcriptomes from the two *P. capsici* isolates infecting pepper allows us to identify molecular functions responsible for the *P. capsici* adaptation to a specific host plant. Our results shed light on how oomycetes interact with various host plants and thus help the identification of targets for plant protection.

## 2 Materials and methods

### 2.1 Pepper host lines, *Phytophthora capsici* isolates, inoculation process, RNA samples

Two lines of pepper (C. *annuum* L.*)* with differing resistance levels to *P. capsici* were used: Yolo Wonder (YW, PM0031), susceptible (S), and Criollo de Morelos 334 (CM334, PM0702), partially resistant (R) (Thabuis et al., 2003; Bonnet et al., 2007). Two weeks after sowing, plantlets were transplanted and grown in the greenhouse for four additional weeks. Two isolates which differed in their level of aggressiveness on pepper were used: isolate Pc107 (called A for adapted) was collected from peppers in the South of France (from INRAE GAFL), and isolate Pc273 (N for non-adapted) was collected from pumpkins in the USA (code LT263 from University of Tennessee).

Prior to inoculation, the apex of 6-week-old pepper plants was removed with a razor blade. Inoculations were performed, as described in Lefebvre and Palloix, (1996), by putting on the wounded stem a 4-mm diameter plug of mycelium, previously grown on a V8 media for 7 days at 22°C. To promote infection, an aluminum square of 4 cm^2^ capped the mycelium plug for three days. Inoculated plants were transferred to a growth chamber at 24°C/22°C temperature on a 12h/12h light/dark cycle. The experiment was triplicated, each triplicate being inoculated with an independent inoculum, to produce three biological replicates. Disease progression was observed on a set of four or six plants per host-isolate interaction at the same conditions as described above. Lengths of stem necrosis were measured at 24- and 72-hours post-inoculation (hpi).

**Figure 1:**
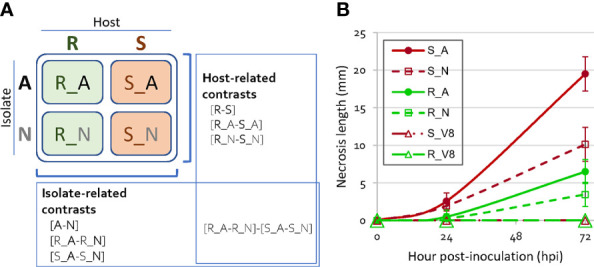
Experimental design of the four host-isolate interactions and disease symptom progression. **(A)** Scheme of the four host-isolate interactions and the seven contrasts considered in DiCoExpress for detecting differentially expressed genes of *Phytophthora capsici*. Three biological replicates were produced per host-isolate interaction. A, adapted isolate; N, non-adapted isolate; V8, mock inoculation; R, resistant host; S, susceptible host. **(B)** Disease symptom progression over 72 hpi on resistant and susceptible peppers inoculated with an adapted and a non-adapted isolates of *P. capsici*. Each dot shows the mean of 16 plants for S and of 24 plants for R. Error bars show the standard deviation. ANOVA test, at 24 hpi p-value=2.28 10^-14^ (R²=0.58), at 72 hpi p-value=4.18 10^-39^ (R²=0.91).

At 24 hpi, twelve total RNA samples were extracted from inoculated plants for the four host-isolate interactions: R_A, S_A, R_N and S_N (**Figure 1A**). Each sample consisted of six pooled stem fragments. The stem fragments are the 5-mm region immediately under the visible stem necrosis. Samples were flash-frozen in liquid-nitrogen and stored at -80°C until RNA extraction. They were ground in liquid nitrogen with a cold mortar and pestle. Total RNA was extracted using QIAGEN Rneasy Plant Mini Kit. RNA-seq libraries were constructed at IPS2 POPS platform (France) by TruSeq_Stranded_mRNA_SamplePrep_Guide_15031047_D protocol (Illumina^®^, California, USA). Sequencing was conducted on an Illumina Hiseq2000 hosted by Genoscope (Evry, France). The RNA-seq samples have been sequenced in paired-end (PE) with a sizing of 260 bp and a read length of 100 bases, lane repartition and barcoding giving approximately 35 million of PE reads per sample (**Supplementary Table S1**).

### 2.2 RNA-seq data trimming and mapping

To facilitate comparisons, raw sequences of the twelve libraries followed the same steps from trimming to count. A preprocessing of quality control was applied, including trimming library adapters and removing PE reads with bad quality (Phred Quality Score < 20, read length < 30 bases). Ribosomal RNA sequences were discarded with the sortMeRNA tool (Kopylova et al., 2012). By using the software STAR (version 2.7.3) with the mode parameters ‘keep the best results’ and ‘adapt intron length from min 5 bp to max 60,000 bp’ (Dobin et al., 2013), the retained PE reads were mapped simultaneously to the genomic scaffolds of the pepper genome of CM334 with annotation Annuum.v.1.6 [35,884 genes, http://peppergenome.snu.ac.kr/, Kim et al., 2017] and to the Phyca11 version of *P. capsici* genome (http://genome.jgi.doe.gov/Phyca11/Phyca11.home.html, Lamour et al., 2012). As further data analyses were restricted to the pathogen gene expression, we built a custom annotation file for the *P. capsici* transcriptome by adding 79 re-annotated CRNs from Stam et al., (2013) and 471 re-annotated RxLRs from Jupe et al., (2013) to the 19,805 transcript models of the *P. capsici* genome, leading to 20,052 non redundant *P. capsici* predicted genes. The abundance of each *P. capsici* gene was calculated with STAR by counting only PE reads that map unambiguously to a single gene, removing multi-hits. Counts were converted into Count Per Million values (CPM*_ik_* = 10^6^ * [number of reads for the gene *i* in the sample *k*/total number of reads for all genes in the sample *k*]).

### 2.3 Identification of differentially expressed genes and co-expressed genes

The full RNA-seq analysis from quality controls to co−expression analysis and differential analysis based on contrasts inside generalized linear models was performed with DiCoExpress, a script-based tool implemented with R language (Lambert et al., 2020). To keep genes with enough mapped PE reads, genes with at least one read in at least two replicates of each host-isolate interaction were filtered. For each selected gene, we applied the Trimmed Mean of M-values (TMM) normalization (Robinson et al., 2010) in order to normalize their number of PE reads counted in the twelve samples. We considered the TMM normalization of counts for each sample suitable for further analysis since the boxplots of normalized counts are similar across the twelve samples (**Supplementary Figures S1A, B**). The principal component analysis of normalized read counts distinguishes samples according to the isolates in both hosts as well as it clusters samples from the same host-isolate interaction, the two first axes explaining 47.6% of the total variation (**Supplementary Figures S1C, D**). The distributions of the number of genes according to their gene expression levels within each library were mostly Gaussian-shaped (**Supplementary Figure S2**), even if the R_A libraries, whose numbers of *P. capsici* PE reads were the smallest, showed left-censored distributions. The expression levels of a gene were homogeneous between replicates of a same host-isolate interaction (**Supplementary Figure S3**). The low number of *P. capsici* PE reads in the samples, compensated by the small variability between replicates, thus only marginally affects the subset of selected genes allowing gene-by-gene comparison to identify the effect of the host plant and of the isolate on the gene expression of *P. capsici*.

To identify among the selected *P. capsici* genes those exhibiting a differential expression (DEG) according to modalities of the biological factors, the TMM-normalized CPM values of each selected gene were compared using the GLM-Poisson (generalized linear models) implemented in the R-package edgeR (McCarthy et al., 2012), by constructing a model including the host (H), the isolate (I), their pairwise interaction (H*I), and the replicate factor (R) which represents the intra-condition heterogeneity: Log_2_(TMM-CPM value) = H + I + H*I + R. The interaction term H*I in the model may reveal meaningful specific interactions between the host and the isolate. DiCoExpress automatically generates a list of contrasts based on the model and provides the DEGs of *P. capsici* when comparing the two host plants (R and S) and the two isolates (A and N) (**Figure 1A**). A gene was considered differentially expressed (DEG) when its Benjamini-Hochberg-corrected (BH-corrected) p-value (False Discovery Rate or FDR) was less than 0.01.

To identify groups of co-expressed genes (CEGs), DiCoExpress uses a Gaussian mixture model based on the normalized expression profiles after an arcsine transformation implemented in the coseq R-package and clusters genes according to their expression profile in all samples.

### 2.4 Gene ontology enrichment analysis

To evaluate the coherence of the results with the biological knowledge, we used the gene ontology (GO) annotation derived from the Phyca11 version of *P. capsici* genome. Of the 20,052 *P. capsici* genes, 9,017 genes (45% of the 20,052 *P. capsici* genes) are annotated, including 471 genes annotated as RxLR and 79 as CRN; 8,467 (42%) are assigned to GO terms. A comparable proportion of the 7,240 selected expressed genes and of the 709 DEGs had GO terms: 4288 (59%) and 443 (62%), respectively. The proportion of CRN and RxLR genes is similar in the set of selected genes (10 and 60, respectively) and in the DEG list (2 and 12, respectively) (**Supplementary Table S2**). The GO enrichment analysis was performed with DiCoExpress (Lambert et al., 2020) in considering only genes assigned to GO terms. GO terms were tested for enrichment or depletion in the lists of DEGSs and CEGs compared to the 7,240 selected *P. capsici* genes, using hypergeometric tests with a p-value threshold of 0.01 implemented in DiCoExpress.

### 2.5 Data accessibility

All steps of the experiment, from growth conditions to bioinformatic analyses, were managed in CATdb database (Gagnot et al., 2008), with the project identifier NGS2013_07_ Pcapsici. This project was submitted from CATdb into the international NCBI repository GEO (Edgar et al., 2002) and SRA under the project identifier GSE206447, which is publicly accessible at https://www.ncbi.nlm.nih.gov/geo/query/acc.cgi?acc=GSE206447.

### 2.6 Reverse transcription quantitative PCR of RxLR effector genes

To confirm changes of the expression pattern observed with RNA-seq analysis, six *P. capsici* genes encoding an RxLR effector were amplified by RT-qPCR. Three *P. capsici* reference genes (*Ubc*, *Ppi2*, *RL13*) were used as constitutive internal controls (Yan and Liou, 2006). Primer pairs were designed with primer3 version 0.4.0 using default parameters (http://primer3.ut.ee/) (**Supplementary Table S3**). Amplification efficiency of each primer pair was calculated based on the slope of the standard curve, using the equation: **E (%) = 100.(-1+10^(-1/slope)^)**. Specificity of primer pairs was validated by BLASTN against *P. capsici* and pepper genomes with parameters adapted for short input sequences, and by analysis of dissociation curves using *P. capsici* and pepper genomic DNA as template. RT-qPCR analyses were first performed on RNA samples collected at 24 and 72 hpi, the 24-hpi samples were the same as those used for RNA-seq analysis. Second, other RNA samples were produced independently with the same method and experimental design from a set of six resistant lines and five susceptible lines sampled at 24 hpi. Three independent biological replicates were produced for each host-isolate interaction. Total RNA was extracted using QIAGEN Rneasy Plant Mini Kit, and treated with Qiagen RNase-Free DNase, following manufacturer instructions. Absence of contaminating DNA was checked by performing a qPCR reaction using the primers of the control pepper gene 'elongation factor 1-alpha EF1’ on RNA samples. Quantification and quality assessment of RNA samples were done with Nanodrop and agarose electrophoresis gel. One µg of RNA was reverse-transcribed to cDNA using the SuperScript III Reverse Transcriptase enzyme kit (Thermo Fisher Scientific) and oligo (dT)18 at 50µM. RT-qPCRs were carried out with the Brillant III ultra-fast SYBR QPCR MM kit (Agilent Technologies) using 1 μL of diluted cDNA (1:10) and primers at 0.2mM each in a reaction volume of 10 μL using the CFX96 Biorad cycler. Each reaction was heated to 95°C for 5 min, followed by 40 cycles of 95°C for 10 s and 64°C for 20 s, and then by a ramp of 0.5°C each 5 s until reaching 95°C. Absence of contamination was checked using two non-template controls per plate. The RT-qPCR amplifications were repeated three times (technical replicates). Quantification of the relative gene expression was performed using the ΔΔCt method (Livak and Schmittgen, 2001).

### 2.7 *In planta* expression of *Phytophthora capsici* RxLR effector genes

We functionally analyzed the effect of ectopic expression of CUST_2407 and CUST_16519 in pepper leaves of resistant and susceptible hosts to *P. capsici* by agro-infection using the PVX-*Agrobacterium*-based transient transformation system (Du et al. 2014).

To evaluate the capacity of *in planta* multiplication and migration of the PVX (used as the T-DNA of the constructs), we assessed the susceptibility of pepper lines to PVX inoculation. The construct pGR106-empty containing PVX was mechanically inoculated on the two cotyledons of six plants from each pepper lines one month after sowing. Two weeks after inoculation, 500 mg from uninoculated apical leaves of each inoculated plant was sampled. Samples were separately ground in a phosphate buffer (0.03M Na2HPO4, 0.2% sodium diethyldithiocarbamate, 4 mL buffer/g of leaves). A double antibody sandwich–enzyme-linked immunosorbent assay (DAS-ELISA) was performed on two batches of three plants per line and absorbance readings of each batch was measured at 405 nm. The virus concentration of each batch was calculated relative to common non-inoculated controls added to the ELISA plate. Finally, a mean relative virus concentration was obtained for each line. All accessions were considered susceptible to PVX as their mean relative virus concentration was greater than three times the mean of non-inoculated controls on the same plate.

As we identified SNPs between isolates A and N for the two RxLR genes, we produced for each gene two constructs (called CUST_2407_A, CUST_2407_N, CUST_16519_A and CUST_16519_N) corresponding to alleles from A and N. Genes without the peptide signal were cloned using primers described in **Supplementary Table S3** and the Gateway technology (Invitrogen, San Diego, CA, USA). Amplicons were transferred into the pDONR_207 by BP-reaction (with gentamycin 25µg/mL) then transferred into the Gateway PVX expression vector pGR106 by LR reaction (kanamycin 50µg/mL). Isolated plasmids for each construct were then introduced by electroporation into *Agrobacterium tumefasciens* (renamed *Rhizobium radiobacter*) strain GV3101 (gentamicin 25µg/mL, rifampicin 50µg/mL, kanamycin 50µg/mL, tetracycline 5µg/mL). We used two control constructs: the pGR106::GFP including the GFP gene was used as a negative control that do not produce HR and to ensure the efficiency of transformation by checking the fluorescence of GFP under blue light in the agro-infected leaves (data not shown), and the pGR106::16240 including the *Phytophthora infestans* gene PITG_16240 (Haas et al., 2009) was used as a positive control as it was demonstrated to trigger HR in pepper (unpublished data).

Each *Agrobacterium* strain was plated on LBA medium (Lysogeny Broth Agar) and grown for 48 hours at 28°C. Bacterial cultures were scraped and resuspended in 10mM MgCl2, 150µM Acetosyringone, 10mM 2-(N-morpholino)-ethane sulfonic acid, adjusted to an OD at 600nm of 4. This bacteria solution was used for agro-infection according to the method described by Du et al. (2014).

We experimentally assessed the response of pepper lines to transient *in planta* expression of the four RxLR constructs in two experiments. Eight week-old seed-grown plants of eleven lines of *C. annuum* with differing levels of resistance to *P. capsici* were grown in greenhouse. For all lines and for each construct to be agro-infected, experiment Exp-1 consisted of piercing four leaves on the same plant six times with a wooden toothpick dipped in the bacteria solution, providing 24 points of infection per host-construct interaction. Experiment Exp-6 consisted of agro-infecting the six constructs separately on the same leaf, by pricking three to four leaves per plant and six plants per line, yielding 18 or 19 infection points per host-construct interaction. We followed the progress of each infection point for 22 days after agro-infection in order to follow the appearance of dark local necrotic lesions at the edge of the infection point or any changes of the leaf. For each interaction between a pepper line and a construct, we reported the percentage of local necrotic lesions in experiment Exp-1 and Exp-6, giving a quantitative assessment of the elicitation of the hypersensitive response (HR).

According to the pepper line and the experiment, we observed from 6 to 88% local necrotic lesions with the positive control construct in the eleven pepper lines. The 461 agro-infections made with the negative control construct did not form local necrotic lesions. Negative control *Nicotiana benthamiana* plants did not show local necrotic lesions around the agro-infection points for all six constructs, ruling out their toxicity and indicating that observed HR are specific to the interaction with pepper. Morover, they showed, with the pGR106::GFP construct, fluorescence under blue light in the apical leaves (Exp-1), showing the efficiency of PVX multiplication and migration in the plant (data not shown). The host R (CM334) reacted strangely to agro-infection, particularly in Exp-6: agro-infected leaves started to turn yellow from 10 days after agro-infection, then began falling 17 days after agro-infection. Results of Exp-1 and Exp-6 are consistent. Transient expression of only one construct per leaf (Exp-1) generally gave higher percentages of local necrotic lesions than agro-infection with all six constructs per leaf (Exp-6), possibly caused by cross-response to several constructs.

## 3 Results

### 3.1 The disease progression differs between the four host-isolate interactions

Two lines of pepper (*C. annuum*), one susceptible (S) and one partially resistant (R), were inoculated with two isolates of *P. capsici*, one adapted (A) and one non-adapted (N) to pepper (**Figure 1A**). Disease symptom progression observed in these four host-isolate interactions diverged slightly in the first 24 hours after inoculation (hpi) (0.3 to 2.6 mm of mean stem necrosis length), then increasingly over time (3.5 to 19.5 mm at 72 hpi, **Figure 1B**). The interaction R_N between the resistant host and the non-adapted isolate showed the smallest mean necrosis length, while the interaction S_A between the susceptible host and the adapted isolate showed the largest. We focused the rest of our analysis on the 24 hpi time point in order to compare gene regulation at an early stage of interaction and at a similar stage of *P. capsici* development to conduct an RNA-seq analysis with sufficient reads to analyze.

### 3.2 The dataset enables the comparison of transcriptional expression of 7,240 genes of *Phytophthora capsici*

After stringent quality assessment and trimming of the twelve libraries corresponding to three biological replicates for each of the four host-isolate interactions, we mapped ~29 to ~41 million paired-end (PE) reads (per library) to the *P. capsici* and *C. annuum* reference genomes, and ~23 to ~32 million PE reads to the gene annotations of both genomes. To focus on gene expression of the pathogen, we considered, for each of the twelve libraries, the 2.6 10^4^ to 1.8 10^6^ PE reads mapped to the 20,052 predicted genes of *P. capsici*, corresponding to 0.11 to 6.97% of the total reads (**Supplementary Table S1**). As expected, libraries from the S_A interaction showed the highest number and percentage of *P. capsici* reads compared to the three other host-isolate interactions (p<0.05, Tukey tests with a Bonferroni correction). The host drastically impacted on the percentage of *P. capsici* reads, the resistant host causing on average a lower percentage than the susceptible one (0.44% of total PE reads from libraries R vs. 3.42% from libraries S, p<0.05, Tukey test). Furthermore, the adapted isolate displayed a higher percentage of expression than the non-adapted (3.43% of total reads from libraries A vs. 0.88% from libraries N, p<0.05, Tukey test). Interestingly, libraries from isolate N did not show a significant difference of read numbers between the hosts (0.63% for R_N and 1.11% for S_N, p>0.05, t-test), while libraries from isolate A showed the extreme values of read number (0.28% for R_A and 5.62% for S_A, p<0.05, t-test). Nearly 80% of the PE reads that mapped to the *P. capsici* genome were unambiguously associated with single copy genes from the 20,052 *P. capsici* annotated genes. A total of 13,540 genes (68%) were expressed with at least one read in at least one sample. The twelve samples had each between 6,170 and 11,434 expressed genes with at least one read. To compare the gene expression level between the four host-isolate interactions, we considered the 7,240 *P. capsici* genes with at least one read in two replicates of each of the four host-isolate interactions (36% of the annotated *P. capsici* genes). These 7,240 genes contained 60 RxLR and 10 CRN genes.

### 3.3 A total of 709 genes of *Phytophthora capsici* are differentially expressed between the four host-isolate interactions

**Figure 2:**
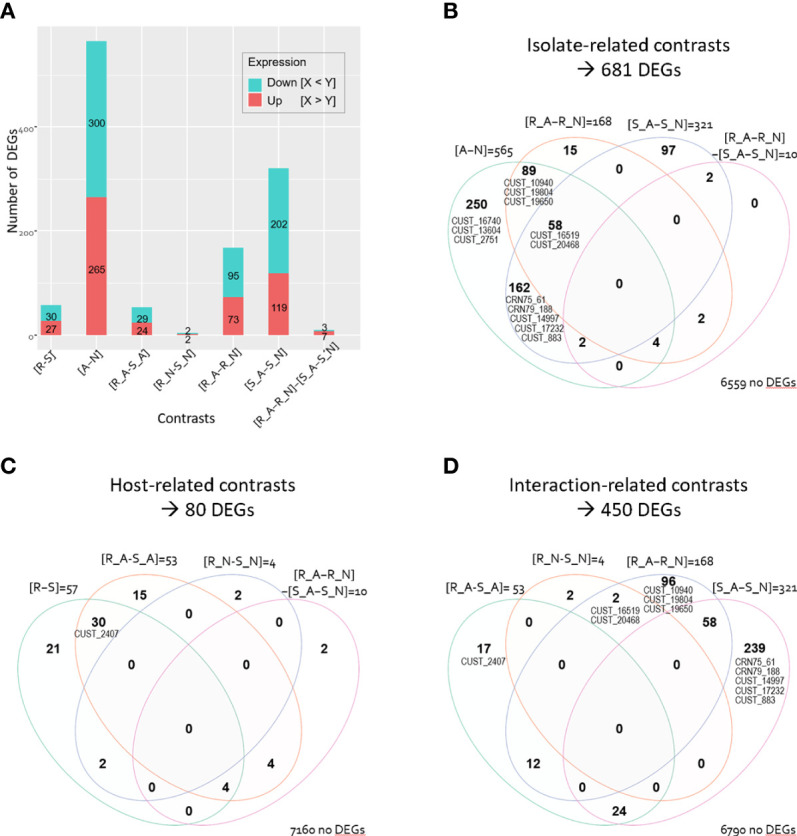
Number of differentially expressed *Phytophthora capsici* genes (DEGs). **(A)** Barplot of number of up- and down-regulated DEGs for the seven contrasts. Venn diagram illustrating the DEG repartition depending on **(B)** the isolate-related contrasts, **(C)** the host-related contrasts, and **(D)** the host-isolate interaction-related contrasts. Venn diagram indicates numbers of DEGs that are common to several contrasts and numbers that are unique to a single contrast. Numbers of genes that are not differentially expressed in the considered contrasts are indicated at the right bottom of each Venn diagram. The CRNs and RxLRs (CUST) are positioned on each Venn diagram in their respective areas.

Out of the 7,240 analyzed genes, we highlighted a total of 709 *P. capsici* genes (9.8%) differentially expressed (DEGs) in at least one pairwise comparison (*Cf*. the seven contrasts in **Figure 1A**; **Supplementary Table S4**). A total of 355 of the 709 genes (50%) were differentially expressed in two or more pairwise comparisons. Globally, we observed a balance number of up- and down-regulated genes for each contrast (**Figure 2A**; **Supplementary Table S5**). However, isolates recovered from the resistant host expressed significantly fewer genes compared to the susceptible host, and genes of isolate A in interaction with pepper expressed significantly fewer genes compared to isolate N.

Comparison of the two isolates revealed the greatest number of DEGs (681 DEGs in the isolate-related contrasts, 9.4% of the 7,240 analyzed genes; **Figures 2A, B**). We identified 565 DEGs between isolates A and N irrespective of the infected host (7,8% in [A-N]). The number of DEGs between A and N was higher when they infected host S than when they infected host R (321 DEGs, 4.4% of the 7,240 genes, in [S_A-S_N]; 168 DEGs, 2.3%, in [R_A-R_N]).

Comparison of the two hosts highlighted only 80 *P. capsici* DEGs (1.1% of the 7,240 genes in the host-related contrasts, **Figures 2A, C**), with 57 host-related DEGs irrespective of the isolate, 53 DEGs specific to isolate A, and only four DEGs specific to isolate N (0.8% in [R-S]; 0.7% in [R_A-S_A], 0.06% in [R_N-S_N]). Noteworthy, only ten genes responded differentially to the host treatment in the two isolates (0.1% in [R_A-R_N]-[S_A-S_N]); those ten genes were also differentially expressed in other comparisons (**Figures 2B, C**). The characterisation of the four host-isolate interactions themselves identified 450 *P. capsici* DEGs (6.2% in the interaction-related contrast, **Figure 2D**).

Among the whole set of 709 DEGs, fourteen genes are annotated as CRN or RxLR (**Supplementary Table S2**). Notably, we identified up to eleven RxLR and two CRN DEGs related to the isolate-contrasts while we found a single RxLR DEG, named CUST_2407, related to the host-contrasts (**Figures 2B, C**). The interaction-related contrasts revealed that nine RxLRs and two CRNs were differentially expressed (**Figure 2D**).

### 3.4 Host-related and isolate-related DEGs belong to different metabolic pathways

The GO enrichment analysis of the set of 2,139 GO terms assigned to the *P. capsici* genome yielded a total of 122 significantly enriched or depleted GO terms within the whole list of 709 DEGs compared to the 7,240 studied genes, with 3 to 50 GO terms according to the considered contrast. Seven, 25 and 90 out of the 122 GO terms were related to cellular components, to biological processes, and to molecular functions, respectively (**Supplementary Tables S4**, **S6**).

#### 3.4.1 Host-related DEGs clearly oppose S_A from the three other host-isolate interactions

The 80 host-related DEGs corresponded to 60 enriched GO terms. DEGs in contrast [R-S] showed significant enrichment in 44 GO terms. Thirty-two were assigned to the parental catalytic activity and mostly included enzymes of class 1 catalyzing redox reactions and of class 3 hydrolyzing various glycosyl bonds. Other GO terms were related to carbohydrate metabolic process, cell proliferation, and transporter activities that enable movement of solutes through the cell membrane. Moreover, two GO terms were related to binding to iron-containing molecules. Contrast [R_A-S_A] revealed eleven specific enriched GO terms, in addition to 34 enriched GO terms shared with the contrast [R-S]. Notably, specific [R_A-S_A]-related DEGs involved transferases (class 2) and isomerases (class 5). Contrast [R_N-S_N] revealed a single additional specific enriched GO term related to the molecular function palmitoyl-(protein) hydrolase activity.

**Figure 3:**
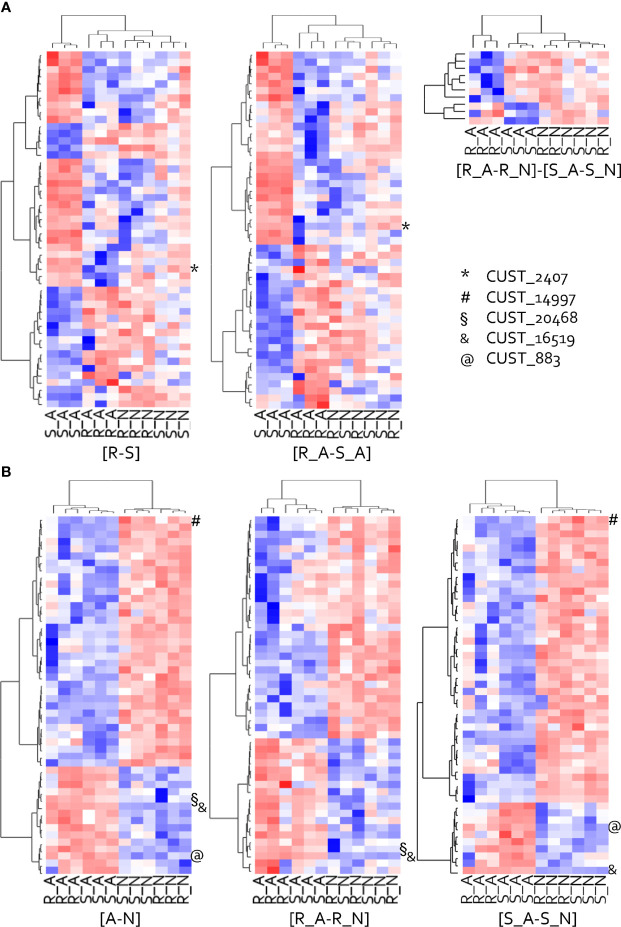
Heat map of the hierarchical clustering of the 50-top host-related **(A)** and isolate-related **(B)** DEGs. Clustering made on behavior patterns of the twelve samples are presented for each studied contrast, except for [R_ N- S_N] that counts only four DEGs. The top DEGs have been ranked on their p-values. Red represents high relative expression and blue represents low relative expression. RxLRs (CUST) are positioned on each heat map.

The hierarchical clustering of the 50 most impacted genes by the host highlighted two major groups of genes with opposing expression patterns in isolate A, whereas their patterns were less clearly differentiated in isolate N (**Figure 3A**). In addition, the S_A interaction had a very specific pattern compared to the other three interactions, which highlighted the specific metabolic performance of the adapted isolate in the susceptible host and the fact that interactions where the pathogen develops poorly had similar gene expressions. Among the 50 most impacted genes, isolate A over-expressed in host S a set of 29 genes, including the RxLR gene CUST_2407. In contrast, isolate A under-expressed 21 genes in S.

#### 3.4.2 Isolate-related DEGs show GO terms depleted in synthesis of nucleic acids and enriched in oxidation of fatty acids

The 681 isolate-related DEGs corresponded to 13 depleted and 57 enriched GO terms. Those GO terms were rather specific to the isolate-related contrasts since only eight of the 57 enriched isolate-related GO terms were common with the 60 enriched host-related GO terms.

The [A-N] contrast showed significant depletion in ten GO terms of which nine were related to synthesis of nucleic acids. Five of them were also depleted in the [S_A-S_N] comparison. Interestingly, contrasting [R_A-R_N] revealed three specifically depleted GO terms related to protein binding and to protein serine-threonine kinase activity.

The [A-N] contrast showed significant enrichment in 40 GO terms. They were related to the ammonium assimilation process involved in the formation of glutamate (glutamic acid), oxidation of the fatty acids, hydrolase activities contained in lysosomes, oxidoreduction reactions, and the ribonucleotide biosynthesis and translation. Intriguingly, contrasting [R_A-R_N] revealed eleven specifically enriched GO terms majorly related to carbohydrate metabolic process, oxidoreductase or hydrolase activities, and the peroxisome.

The hierarchical clustering of the 50 most impacted DEGs for contrast [A-N] revealed two groups of genes with contrasted expression patterns among the twelve samples (**Figure 3B**). Those 50-top isolate-related DEGs that clearly opposed A and N, showed similar patterns in hosts R and S. One group of 35 DEGs, including the RxLR gene CUST_14997, were under-expressed by isolate A and over-expressed by isolate N. The other group of fifteen DEGs, including three RxLR genes (CUST_20468, CUST_16519, CUST_883), showed a clear opposite behavior.

### 3.5 *Phytophthora capsici* DEGs split in two clusters of co-expressed genes with an opposite pattern between isolates

**Figure 4:**
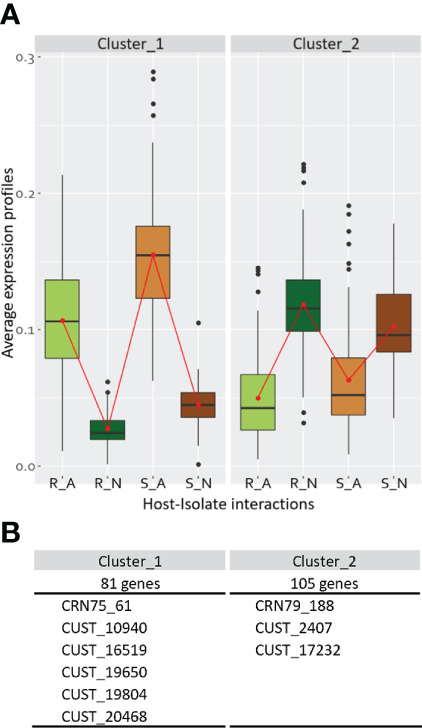
Co-expression clusters for the *Phytophthora capsici* DEGs issued from the interaction-related contrasts. For each cluster, are indicated **(A)** the average expression profiles of the four host-isolate interactions, and **(B)** the size of co-expression clusters with the list of included CRN and RxLR (CUST) genes within the cluster. Data results from the analysis of the 450 differentially expressed genes (DEGs) from the union of the four interaction-related contrasts.

The co-expression analysis of the twelve biological samples based on the average expression profile of the 450 DEGs belonging to the interaction-related contrasts revealed two clusters of co-expressed genes (CEGs) (**Supplementary Table S7**; **Figure 4**). Cluster 1, containing 81 genes including one CRN and five RxLRs, grouped together DEGs strongly expressed by isolate A compared to isolate N. It is enriched for 30 GO terms of which five GO terms were related to transferases recruited for the formation of ornithine. Nine GO terms were related to hydrolases, implied in replication and repair of nucleic acids and in cell cycle regulation, and eight GO terms belonged to ligase activities that catalyze the formation of fatty acids. Cluster 2, composed of 105 genes with one CRN and two RxLRs, showed the opposite pattern, strongly expressed by isolate N compared to isolate A. It was depleted for one molecular function GO term related to nucleic acid binding. Twenty GO terms were enriched: six were related to the digestion of non-functional molecules by hydrolases occurring in the lysosome, five included transferase-related functions from which some could be involved in sphingolipid metabolic process and in methanogenesis, and three GO terms were involved in redox reactions (**Supplementary Table S8**).

### 3.6 Isolate A under-expresses RxLR CUST_2407 specifically in host R, while isolates A and N over-express RxLRs CUST_16519 and CUST_14997, respectively, irrespective of the host

**Figure 5:**
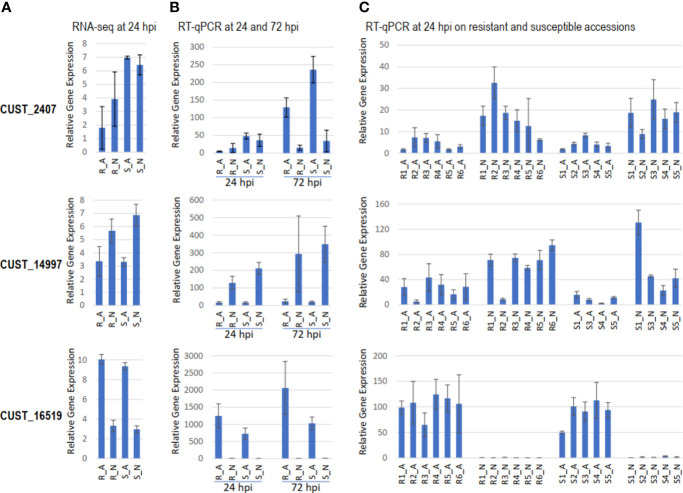
Gene expression level of RxLR genes CUST_2407, CUST_14997 and CUST_16519 in different host-isolate interactions. Barplot of **(A)** the TMM-normalized gene expression measured by RNA-seq in hosts R and S at 24 hpi. Barplot of gene expression measured by RT-qPCR relative to the expression of the housekeeping gene *Ubc* **(B)** in hosts R and S at 24 and 72 hpi, and **(C)** in six resistant lines [R1 (PM0217/PI201234), R2 (PM0659/Perennial), R3 (PM0702/CM334, R), R4 (PM1407/Phyo 636), R5 (PM1409/Vania), R6 (PM1686/Breeding Line B2)] and five susceptible lines [S1 (PM0031/Yolo Wonder, S), S2 (PM0076/Doux Long des Landes), S3 (PM0807/H3), S4 (PM0867/(Meskix872)AP1-B1), S5 (PM0972/Quadrato d’Asti rouge)] of pepper at 24 hpi. The same profiles of expression measured by RT-qPCR was observed with the reference genes *Ppi2* and *RL13*.

By RT-qPCR, we validated the expression variation of six RxLR effector genes, three DEGs and three non-DEGs according to RNA-seq analysis, in hosts R and S and in a set of eleven hosts differing for resistance to *P. capsici* (**Figure 5**). The three DEGs chosen were the most contrasted RxLRs between host-isolate interactions. CUST_2407, the single RxLR DEG in contrast [R-S], was more expressed in S (FDR=9.56 10^-8^). CUST_16519 and CUST_14997, the two most RxLR DEGs in contrast [A-N], were more expressed in A (FDR=4.58 10^-57^) and in N (FDR=1.20 10^-13^), respectively. At 24 hpi, RT-qPCR profiles in the four host-isolate interactions corroborated the RNA-seq expression profile, confirming the accuracy of RNA-seq data analysis. CUST_2407 exhibited contrasting expressions in isolate A between hosts R and S, CUST_14997 and CUST_16519 exhibited contrasting expressions between isolates A and N, while CUST_5407, CUST_15481 and CUST_17572 (for which RNA-seq expression level did not vary) did not show significant differential RT-qPCR expression (**Figures 5A, B**). Comparison of RT-qPCR at 24 and 72 hpi highlighted the time-course of gene expression along the *P. capsici*-pepper interactions. Isolate A under-expressed CUST_2407 in R compared to S at 24 hpi as well as 72 hpi, while isolate N did not differentially express it when infecting R and S and did not increase compared to 24 hpi. Isolate N over-expressed CUST_14997 at the two time-points in both hosts and slightly increased its expression along the time course. Isolate A over-expressed CUST_16519 in both hosts and increased its expression along the time-course (**Figure 5B**).

Considering our analysis conducted on the set of six resistant lines and five susceptible lines to *P. capsici*, we found that the expression profiles of CUST_14997 and CUST_16519 were similar to profiles observed in the R_A, R_N, S_A and S_N interactions. In contrast, the profile of CUST_2407, that was poorly expressed at 24 hpi, did not fully corroborate the previously observed differential expression between hosts R and S (**Figure 5C**). This small discrepancy between both independent RT-qPCR experiments may be due to a difference of kinetics, of time points of sampling, and of plant physiological status between the both experiments, since both experiments were performed at different periods of the year. The independent experiment done on the set of eleven lines suggested isolate A generally under-expressed CUST_2407 and CUST_14997, while it generally over-expressed CUST_16519 compared to N. The strong expression of CUST_16519 by A may aid the progression of *P. capsici in planta*, while its weak expression by N may slow it down. On the contrary, the weak expression of CUST_2407 and CUST_14997 by A may be due to their recognition by the host plant preventing the progression of isolate A. The three effectors CUST_2407, CUST_14997 and CUST_16519 appear thus to be involved in the adaptation of *P. capsici* to infect pepper.

### 3.7 Transient expression of RxLRs CUST_2407 and CUST_16519 triggers HR at low frequency in susceptible host

**Figure 6:**
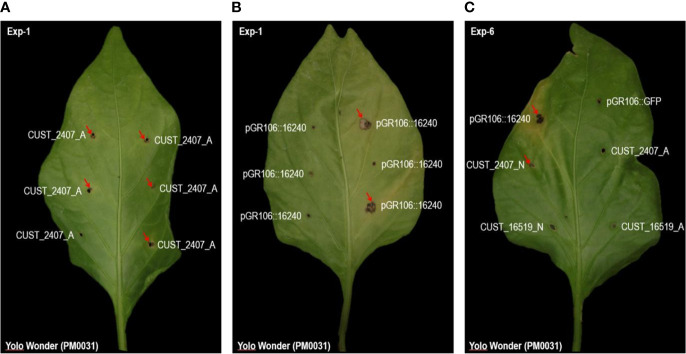
Up-side of host S leaves agro-infected by the RxLR constructs and the control constructs in Exp-1 and Exp-6 experiments. In Exp-1, four leaves of each pepper accession were agro-infected at six points per leaf with a single construction. In Exp-6, 18 or 19 leaves of each accession were agro-infected with four RxLR constructs and two controls separately on each leaf. Exp-6 thus consisted to agro-infect each RxLR construct with the positive and negative controls on the same leaf. Constructs pGR106::GFP and pGR106::16240 were used as negative and positive controls, respectively. The 460 agro-infected points performed on the eleven pepper accessions with the negative control produced no hypersensitive response (HR). The 464 agro-infected points performed on the eleven pepper accessions with the positive control yielded an average of 36,4% HR. The four other four constructs corresponded to the RxLR genes CUST_2407 and CUST_16519 isolated from isolates A and N. The host accession is Yolo Wonder (PM0031), susceptible to *Phytophthora capsici*. Red arrows indicate the dark local necrosis observed around the agro-infection point, scored as a HR. **(A, B)** illustrate the quantitative efficiency of transient *in planta* expression of CUST_2407_A and pGR106::16240, respectively, justifying the need for multiple agro-infection points per construct. **(C)** illustrates the consistency between Exp-1 and Exp-6 experiments, as shown here for the RxLR CUST_2407_A and the control pGR106::GFP, and as reported in **Supplementary Table S9**. Photographs were taken at 20 days after agro-infection.

**Figure 7:**
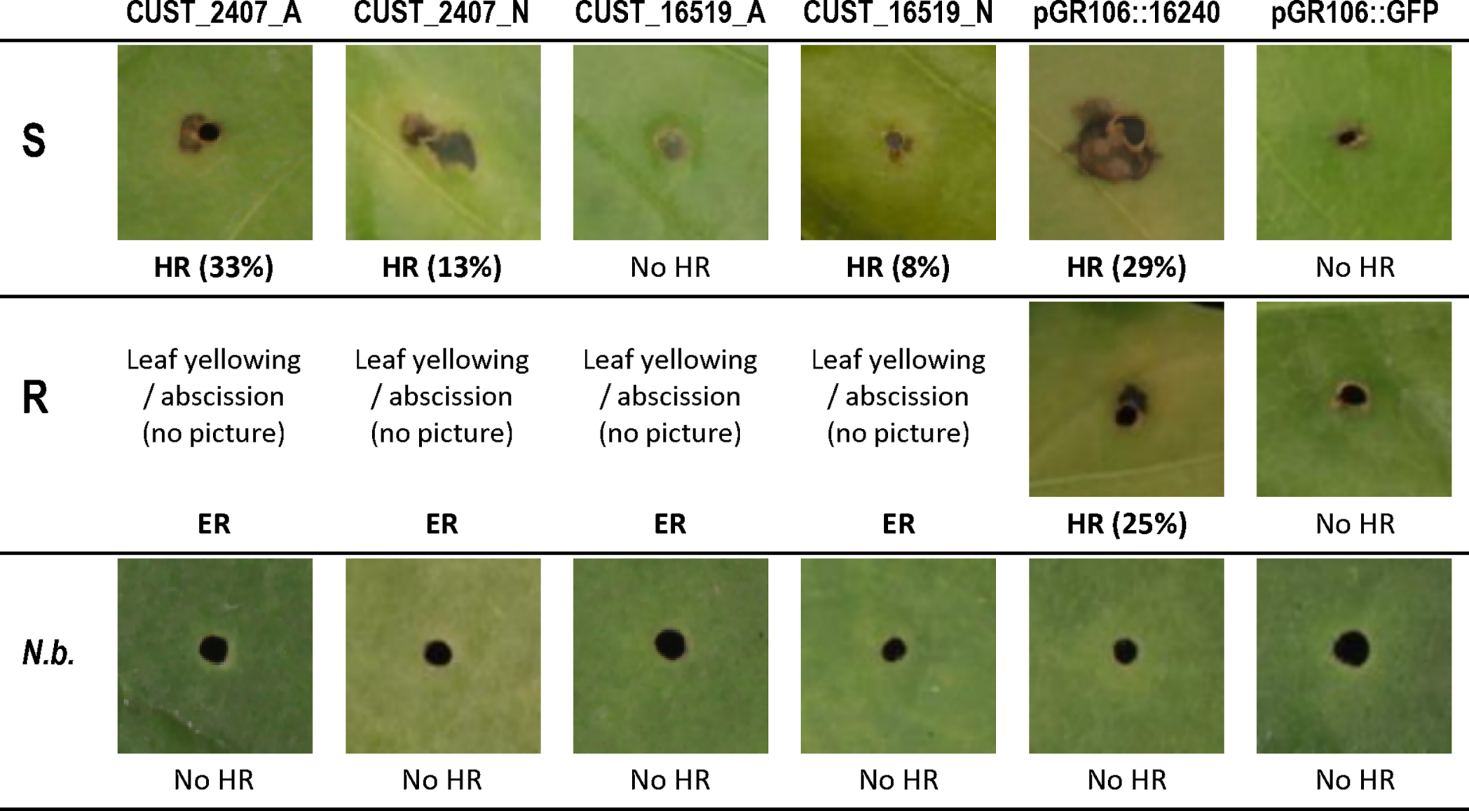
Symptoms of transient *in planta* expression of RxLR genes CUST_2407 and CUST_16519 from isolates A and N in leaves of pepper hosts R and S after PVX agro-infection. Dark local necrosis around the agro-infection points was scored as a hypersensitive response (HR). With constructs CUST_2407 and CUST_16519 from isolates A and N, host R showed yellowing followed by abscission of agro-infected leaves, which were assumed to correspond to extreme resistance (ER). In parentheses are indicated the percentages of 24 agro-infected points which showed a HR. The constructs pGR106::16240 and pGR106::GFP were used as positive and negative controls, respectively. Leaves of *Nicotiana benthamiana* (*N.b.*) do not show dark local necrosis with the six agro-infected constructs. Photographs were taken at 15 to 20 days after infection.

We first developed an efficient method for transient expression of RxLRs in pepper (**Figure 6**), and then investigated by transient *in planta* expression how effector CUST_2407, the only RxLR DEG between hosts, and effector CUST_16519, the most strongly RxLR DEG between isolates, interact with resistant and susceptible pepper lines. We observed that transient expression of the CUST_2407 constructs derived from isolates A and N in host S (Yolo Wonder) triggered local necrotic lesions at a low frequency: 33% and 13%, respectively (in Exp-1). Conversely, in host R (CM334), we did not record any lesions but agro-infected leaves turned yellow and several of them detached. Further, CUST_16519_N (cloned from isolate N) induced some necrotic lesions in host S whilst CUST_16519_A did not produce a visual response in host S. In host R, no lesions were observed with either construct but leaves detached (**Supplementary Table S9**; **Figure 7**).

The transient expression results thus confirmed the above DEG analysis. Hosts R and S differentially responded to the *in planta* expression of CUST_2407 (indistinctly from A and N), that triggers a few HR local necrotic lesions in S and leaf abscission in R. Only host S produced an HR in response to the *in planta* expression of the CUST_16519_N construct.

### 3.8 RxLRs CUST_2407 and CUST_16519 highlight specific recognitions in susceptible and resistant pepper germplasm

In addition to hosts S (Yolo Wonder) and R (CM334), constructs CUST_2407 induced local necrotic lesions in nine other germplasm accessions, with coherent results between the two experiments (**Supplementary Table S9**). Notably, CUST_16519_A and CUST_16519_N triggered high percentages of HRs in the lines PM1686 (resistant to *P. capsici*) and PM0972 (susceptible to *P. capsici*) (**Supplementary Figure S4**). For the two RxLR effectors, we did not reveal any association within the eleven lines tested between the resistance level to *P. capsici* and the percentage of HRs, irrespective of the constructs.

## 4 Discussion

**Figure 8:**
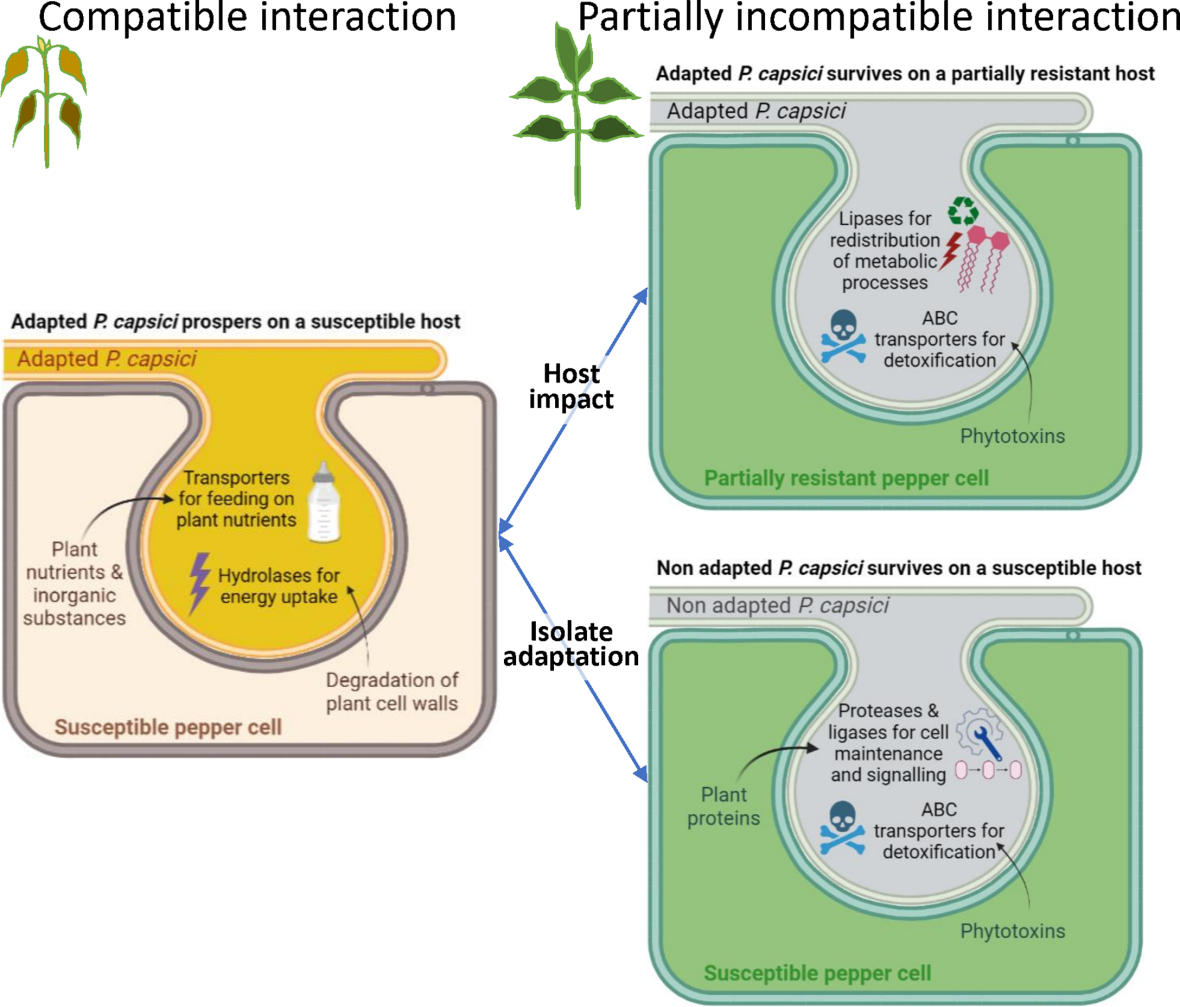
Simplified model of major molecular mechanisms in pepper – *Phytophthora capsici* interaction based on analysis of RNA-seq data at 24 hpi. The S_A interaction between the susceptible host and the adapted isolate leads to a compatible interaction that allows *P. capsici* to thrive by feeding on plant nutrients and absorbing energy produced by the degradation of plant cell walls, through exchanges that would occur in the haustorium. When the host is partially resistant or the isolate is non-adapted, this leads to a partially incompatible interaction, such as interactions R_A, S_N or R_N. In the R_A interaction, *P. capsici* recycles its own resources through lipid metabolism and detoxifies phytoalexins to strive for its survival. In the S_N interaction, *P. capsici* also detoxifies phytoalexins and activates protein metabolism for maintenance of its own cells. R_N is not represented here since our study revealed only four genes that are differentially expressed with S_N. This tentative model simply presents the hypotheses resulting from our study and is destined to be completed or modified to tend towards the complexity of the natural system, thanks to future analyses carried out by the whole scientific community.

The outcome of this transcriptomic analysis of *P. capsici* during its interaction with pepper provides valuable insights into *(i)* how the host plant impacts the expression of pathogen genes at the very beginning of infection, and *(ii)* how the adaptation of a pathogen to a host depends on its gene expression. For addressing these two crucial questions, we measured the gene expression of two isolates of *P. capsici* (A and N) differing in their level of virulence (synonym here of aggressiveness) on two pepper lines (R and S) which differ for their spectrum of immunity to *P. capsici*. Comparing the four host-isolate interactions revealed 702 *P. capsici* DEGs (**Figure 2**). We observed the greatest number of DEGs by contrasting isolates. Host-related and isolate-related DEGs primarily belong to different GO terms suggesting differentiation in the metabolic pathways of the pathogen that has been impacted by the host and those that are mobilized for the pathogen adaptation (**Figure 8**).

### 4.1 The adapted *Phytophthora capsici* isolate mobilizes its transporters to feed on the susceptible host

Contrasting the adapted (A) and the non-adapted (N) isolates revealed 681 DEGs including eleven RxLR and two CRN effectors. The major difference between isolates A and N resides in their perception of the plant species. Isolate N exhibits minimal growth on pepper regardless of the level of resistance, and its gene expression remains limited being unable to metabolize pepper or so as not to exhaust its resources. The total number of Illumina RNA-seq reads in N was equivalent in both hosts. However, the number of isolate-related DEGs is twice as high when isolates infect the susceptible host (S) compared to when they infect the resistant host (R), suggesting that on host S the two isolates behave almost like different microorganisms, while host R reduces isolate A’s metabolism to the status of isolate N.

Interestingly in both hosts, isolate A in comparison to N reduces expression of its genes involved in synthesis of nucleic acids and increases expression of genes coding transporters, suggesting a key role of these two pathways in the adaptation of a *P. capsici* to infect pepper. In host S, the isolates notably differed in their expression of genes associated with transport of nutrients and inorganic substances, detoxification process and protein metabolism (**Figure 8**). The higher expression of transporters of nutrients and inorganic substances by isolate A might support its growth, as proposed by Abrahamian et al. (2016) in the compatible interaction between *P. infestans* and its tomato and potato hosts. In contrast, isolate N over-expressed genes encoding for ATP-binding cassette (ABC) transporters and genes related to protein metabolism, particularly genes coding putative serine proteases without signal peptide and putative ubiquitin ligases (**Supplementary Table S6**). ABC transporters, known to be involved in detoxification processes and required for organ growth, nutrition requisition, and development, may protect *P. capsici* against natural toxic compounds produced by the susceptible host. Moreover, the gene family of ABC transporters have significantly expanded in the most aggressive isolates of *P. capsici*, supporting that ABC transporters play also a role of virulence-associated effectors during *P. capsici* evolution such as RxLR and CRN effectors (Lee et al., 2021). Zuluaga et al., (2016b) also reported high levels of transcript accumulation of *P. infestans* ABC transporters during its biotrophic phase with a susceptible tomato cultivar. Non-secreted serine proteases are involved in a variety of intracellular processes including signal peptide processing and vacuole maintenance (Muszewska et al., 2017). Those genes involved in protein turnover may support the development of isolate N in host S. Ubiquitin ligases are involved in the degradation of proteins *via* the proteasome, and those of N might disturb immunity in host S. This is reminiscent of bacterial type III effectors exhibiting E3 ubiquitin ligase activity *in planta*, manipulating host cell processes and inducing plant cell death (Magori and Citovsky, 2011; Singer et al., 2013; Maculins et al., 2016).

The insignificant number of host-related DEGs for isolate N could be equated with the fact that N is poorly adapted to use pepper as a food source, as if it survives on a minimum medium. Isolate N was indeed originally isolated from a pumpkin. This observation raises the question of whether there may be special forms (or *formae speciales*) in *P. capsici*, which reflect the ability of the isolates to infect a specific plant species as reported in several fungi. Such diversification could, for example, be driven by epigenetic changes or sexual recombination events followed by host-selection for the most adapted variants. Specialization of a pathogen to a host depends on its ability to suppress host defense and to mobilize nutrients to feed on the host. Isolate A probably manipulates the cell machinery of pepper at an early stage of the infection while isolate N is unable to access the nutrients. The isolate-related DEGs are thus candidate genes for adaptation of *P. capsici* to the pepper host, such as suggested when comparing the gene transcriptional changes between *Zymoseptoria tritici* during its interaction with compatible hosts of the species *Triticum* and a *Brachipodium* nonhost (Kellner et al., 2014).

### 4.2 *Phytophthora capsici* recycles its own resources to survive on the partially resistant host

The number of *P. capsici* DEGs when comparing the hosts is much lower than when comparing the isolates as we found only 80 host-related DEGs. Host-related differential expressions are mostly observed with the adapted isolate (**Figure 8**). Moreover, we observed globally more down-regulated genes when isolates infect host R than when they infect host S.

In host S, isolate A majorly over-expressed genes encoding transporters of nutrients and inorganic substances, and genes related to carbohydrate metabolism, particularly encoding 'hydrolase activity acting on glycosyl bonds'. Again, transporters of nutrients and inorganic substances might facilitate development of isolate A by feeding nutrients from host S. Hydrolysis of glycosidic bonds is crucial for energy uptake, cell wall expansion and degradation, and turnover of signaling molecules. The corresponding enzymes may degrade the plant cell wall and supply energy to help *P. capsici* thrive in pepper, as is frequently observed in compatible interaction with necrotrophic pathogens (Sprockett et al., 2011).

Conversely in host R, isolate A over-expressed genes related to lipid metabolism and genes encoding ABC transporters. Strong expression of genes encoding ‘phospholipase D alpha’, secreted at the outside layer of the host cell plasma membrane and involved in the synthesis of phosphatidic acid (PA) mediates signal transduction in many cellular processes (Meijer et al., 2011). This suggests that in order to subsist on host R, isolate A might induce a redistribution of the metabolic processes associated with energy production from glycolysis to lipid metabolism in response to the inhibition of cell wall biosynthesis. This pathway is frequently mobilized in organisms that are in a state of starvation to compensate for the absence of foods, such as *P. capsici* which adapts to peppers treated with fungicides (Pang et al., 2016). *P. infestans* phospholipases are also implicated in pathogenicity (Meijer et al., 2011) and are associated with the transition from biotrophy to necrotrophy in the compatible interaction between tomato and *P. infestans* (Zuluaga et al., 2016a). Additionally, ABC transporters may be required for virulence by detoxifying phytoalexins produced by host R during its infection by isolate A (Loisel et al., 2016).

### 4.3 RxLR CUST_2407 triggers host resistance in CM334 and RxLR CUST_16519 helps *Phytophthora capsici* adapt to pepper hosts

RxLR effectors were described to manipulate host cell function, either to facilitate infection (RxLR is thus a virulence factor) or to trigger defense responses (as an avirulence factor) (Vleeshouwers et al., 2011). *P. capsici* effectors were determined among DEGs. In total, our RNA-seq analysis highlighted fourteen putative secreted effectors corresponding to DEGs between the four host-isolate interactions: twelve were classified as RxLR effectors and two as CRN effectors (**Supplementary Table S2**; **S7**). Globally, RxLRs are more frequently over-expressed by isolate A than by isolate N, while isolate N over-expressed a few RxLRs only when it infects host S. Given that RxLRs modulate the host physiology to allow the pathogen to infect it, the low expression of RxLRs by N might explain its difficulty to colonize pepper hosts.

Our study identified a single RxLR gene whose expression varies depending on the host plant: RxLR CUST_2407 was under-expressed when the adapted isolate A infects the resistant host R and over-expressed when it infects the susceptible host S. Similar, expression-based adaptation to resistance has been described for the *P. infestans* RxLR effector Avr-vnt1 where the gene is only fully expressed in plants containing the cognate resistance gene, *Rpi-vnt1*, after the biotrophic phase and expression is weak or absent in the early stages of the infection (Stefańczyk et al., 2017). The mechanism of effector-triggered immunity (ETI) thus partly supports the expression pattern of RxLR CUST_2407. Once the pathogen introduces this effector into the resistant plant cells, a plant resistance protein would recognize it. This recognition would then trigger the plant immunity reinforced by the activation of defenses, reducing the pathogen development, and consequently, reducing the CUST_2407 expression level in R compared to S. The transient *in planta* expression of CUST_2407_A (cloned from isolate A) yielded the yellowing of infected leaves of host R (CM334). This process consequently causes the abscission of the leaf (removing the infected tissues). We also observed leaf abscission in CM334 whereas there was no abscission but spread of a large necrosis in Yolo Wonder when infiltrated with a solution of *P. capsici* zoospores (data not shown). In Arabidopsis, a protein belonging to the family of transcription cofactors NPR1 (nonexpressor of pathogenesis-related genes 1) was shown to be responsible for the cell differentiation in the abscission zone and to be a positive regulator of systemic acquired resistance in plants as a receptor of salicylic acid and inducing defense genes (Olsson and Butenko, 2018). In the pathosystem Arabidopsis-*Pseudomonas syringae*, the leaf abscission was described as a defense mechanism triggered by effectors (Patharkar et al., 2017). Absence of local necrotic lesions in CM334 might be due to an early and rapid elicitation of extreme resistance (ER) response that arrests the hyphal growth and that prevents the appearance of local necrotic lesions, as suggested for the model *Nicotiana benthamiana*-PVX (Bendahmane et al., 1999). Transient *in planta* expression of pathogen proteins generally enhances the plant response compared to a natural infection. In contrast, the transient *in planta* expression of CUST_2407_A in the susceptible host Yolo Wonder produced few cases of local necrotic lesions, suggesting its insufficient recognition by this plant accession and therefore allowing the development of the pathogen. Finally, we hypothesize that CUST_2407 may play the role of an avirulence gene in CM334. The local necrotic lesions triggered by the *P. capsici* effector CUST_2407 on susceptible plants may reflect the establishment of a weakly active, but ineffective, defense mechanism, unable to halt the infection of Yolo Wonder by *P. capsici*.

The RxLR CUST_16519 elicited different responses according to the isolate. Isolate A over-expressed it at 24 hpi irrespective of the host, and its expression increased at 72 hpi. In contrast, isolate N drastically under-expressed CUST_16519 in tested hosts throughout the time-course. The strong expression of CUST_16519 by A would inactivate the plant defense and promote the progression of *P. capsici in planta*, while its weak expression by N would allow the defense mechanisms to slow it down. The few local necrotic lesions triggered by CUST_16519_N in the susceptible host Yolo Wonder indicates its weak effector-plant receptor recognition. Again, this specific interaction between CUST_16519_N and Yolo Wonder is not sufficiently effective to prevent infection, and CM334 escapes infection through the senescing of leaves. Finally, we hypothesize that CUST_16519_A may play the role of a virulence gene in Yolo Wonder.

In addition, we identified two other specific host-construct interactions. RxLR CUST_16519_A induced a high percentage of local necrotic lesions (of the same order as the positive control) in the resistant line PM1686, and RxLR CUST_16519_N induced a high percentage of local necrotic lesions in the susceptible line PM0972. These contrasting responses suggest that CUST_16519 is not a major determinant of the general response of pepper to *P. capsici* infection. This first analysis of *in planta* interactions between RxLRs and the pepper gene pool deserves to be extended to the wide diversity of *Capsicum* spp. maintained in genebanks (Tripodi et al., 2021) and to a large repertoire of RxLRs. A high degree of genetic diversity in *P. capsici* populations, evidence of population outcrossing and sufficient migration, has been described in several geographic regions, such as the Central States of Mexico (Castro-Rocha et al., 2016). The recent genome resequencing of Mexican isolates from different host species revealed more than 2,100 unique host-specific RxLRs and CRNs that could determine the adaptation capacity of *P. capsici* (Reyes-Tena et al., 2019).

Our experiments provide a quantitative assessment of the elicitation of the hypersensitive response. However, transient gene expression is reputed to be challenging in pepper, which raise non-specific defense responses to inoculation with *A. tumefaciens* (Lee et al., 2004). This is why we preferred to use PVX-mediated agro-infection that limits the number of bacteria in contact with plant providing more reproducible results (data not shown). Agro-infection consists of delivering the PVX genome including targeted RxLR effectors *via Agrobacterium* into plants. Then, *Agrobacteria* translocate the T-DNA into plant cells around the wound, and the PVX further spreads into adjacent cells and expresses the RxLR gene. We demonstrated that the tested pepper lines were all susceptible to PVX, even if we observed variability in response to PVX infection that may modify the efficiency of agro-infection. To assess host response to an RxLR construct, we also favored agro-infections with only one construct per leaf to avoid the risk of cross-reactions that can occur when different constructs are agro-infected on the same leaf. Nevertheless, the observed percentages of local necrotic lesions with the positive control pGR106::16240 testified to a response variability between the host lines, which can still hamper the interpretation of the results.

## 5 Conclusion

To conclude, we demonstrate that the host plant has a significant impact on the gene expression of an adapted pathogen during its early stage of infection. Mainly, the development of the pathogen on the host and its gene expression depend on its ability to mobilize food that, in turn, modifies the gene-regulatory program of the pathogen. Taking together, our results suggest that *P. capsici* may be subjected to nutrient limitation impeding its development, either for the two isolates on the resistant host, or for the non-adapted isolate on pepper in general.

We also showed that different survival strategies exist between an adapted isolate and a non-adapted isolate of the same pathogen species to develop on the plant. The gene expression of the non-adapted isolate is invariable on the potential host (irrespective of the R or S configuration), while the adapted isolate feeds on the susceptible host or develops starvation-survival responses in the resistant host (**Figure 8**).

Moreover, our results suggest two RxLR effectors, CUST_2407 and CUST_16519, are differentially expressed according to host or isolate, respectively, and appear to play a minor role in triggering the quantitative resistance of pepper to *P. capsici*. Their weak effect on inducing a host defense may result from a limited affinity between the pathogen effectors and the corresponding plant receptors, that could explain the partial resistance. This result limits their use in an effectoromic screen of pepper germplasm to identify new durable resistance sources.

However, our results suggest different ways to promote plant health. Genes responsible for synthesis of plant nutrients mobilized by pathogens during *in planta* biotrophic growth stage may become targets for plant defense improvement. Breeders could select genes that reduce carbohydrate compounds in plants, as long as there is no negative effect on the plant development, and that induce production of antifungal phytoalexins not recognized by detoxifying transporters such as ABC transporters of *P. capsici*. Candidate proteins for the adaptive response of *P. capsici* to different host species may assist development of novel fungicides. Chemists could use this approach to identify drug molecules that cannot be excluded by the pathogen’s ABC transporters.

## Data availability statement

The datasets presented in this study can be found in online repositories. The names of the repository and accession number can be found at https://www.ncbi.nlm.nih.gov/geo/, GSE206447.

## Author contributions

VL conceived, designed and coordinated the research. AM, SB, AB, VL, ES, BC and NT carried out wet-lab experiments at INRAE GAFL and INRAE IPS2. VL, VB, GM, GR, and JC performed bioinformatic and statistical analyses. VL, GM, ES, BC, IH and KL contributed to data interpretation. GM presented the raw results and a primary interpretation in his PhD manuscript. VL wrote the submitted manuscript and prepared figures, tables and **supplementary material**. All authors contributed to manuscript revision, read, and approved the submitted version.

## Funding

This work was supported by INRAE Plant biology and breeding Division (EFFECAPS project), Agropolis Fondation (Protéines phytopathogènes 1300-002 project) and French National Research Agency (EFFECTOORES ANR-13-ADAP-0003 project). The funders had no role in study design, data collection and analysis, decision to publish, or preparation of the manuscript.

## Acknowledgments

We are grateful to Drs. Odile Berge, Marie-Laure Martin-Magniette and Etienne Delannoy for their valuable advice and helpful discussions of this work. We acknowledge Dorine Achard and Marion Szadkowski for technical assistance for the cloning of effector genes in PVX binary vector and for advices for assessing susceptibility level of pepper lines to PVX, respectively, the INRAE GAFL’s genebank CRB-Lég as supplier of seeds for pepper accessions used in this study, members of the INRAE GAFL’s plant culture team that took care of plants, and Dr. Sean Chapman, who kindly supplied the Gateway PVX expression vector pGR106 used in transient expression experiments. KL received a fellowship from Agropolis Fondation to spend a sabbatical year at INRAE GAFL. GM received a doctoral scholarship (SOLEFFECT project) from INRAE Plant biology and breeding Division (50%) and French Provence-Alpes-Côte d’Azur Region (50%), with the support of breeding companies Gautier Semences and Rijk Zwaan France. The authors declare no conflict of interest.

## Conflict of interest

The authors declare that the research was conducted in the absence of any commercial or financial relationships that could be construed as a potential conflict of interest.

## Publisher’s note

All claims expressed in this article are solely those of the authors and do not necessarily represent those of their affiliated organizations, or those of the publisher, the editors and the reviewers. Any product that may be evaluated in this article, or claim that may be made by its manufacturer, is not guaranteed or endorsed by the publisher.

## Supplementary material

Supplementary Figure 1 Data quality control of the twelve sample libraries. Boxplot of the library sizes for each sample **(A)** before and **(B)** after the TMM-normalization, respectively. First and second axes of the Principal Component Analysis (PCA) **(C)** on the raw and **(D)** normalized counts. A, adapted isolate (Pc107); N, non-adapted isolate (Pc273); R, resistant host (CM334); S, susceptible host (Yolo Wonder). R1, R2 and R3 are samples from the three independent replicates. On PCAs, the three dots of a same color correspond to three independent replicates for a specific host-isolate interaction.

Supplementary Figure 2 Gene distribution by sample according to their expression level. The level of expression for a gene is expressed by the log-transformation of its TMM-normalized count per million values (TMM-CPM). The three biological replicates (Rep) are represented in column. The four host-isolate interactions are represented in line. A, adapted isolate (Pc107); N, non-adapted isolate (Pc273); R, resistant host (CM334); S, susceptible host (Yolo Wonder).

Supplementary Figure 3 Projection of the mean gene expression of a host-isolate-interaction (y-axis) on the gene expression of a single replicate from the same host-isolate interaction (x-axis). The level of gene expression is expressed by the log-transformation of its TMM-normalized count per million values (TMM-CPM). The projections of the three biological replicates (Rep) are represented in column. The four host-isolate interactions are represented in line. A, adapted isolate (Pc107); N, non-adapted isolate (Pc273); R, resistant host (CM334); S, susceptible host (Yolo Wonder).

Supplementary Figure 4 Symptoms of transient *in planta* expression of RxLR genes CUST_2407 and CUST_16519 from isolates A and N in leaves of seven resistant and four susceptible pepper lines after PVX agro-infection. Red arrows indicate the dark local necrosis observed around the agro-infection point, scored as a hypersensitive response (HR). For each host-RxLR combination, HR on the right side of the photograph means that a HR was observed around at least one infection point, in either the Exp-1 or the Exp-6 experiment. A dot means that no HR was observed. With constructs CUST_2407 and CUST_16519 from isolates A and N, host CM334 (R in the manuscript) showed yellowing followed by abscission of agro-infected leaves, which were assumed to correspond to extreme resistance (ER). The constructs pGR106::16240 and pGR106::GFP were used as positive and negative controls, respectively. Leaves of *Nicotiana benthamiana* did not show dark local necrosis with the six agro-infected constructs. Photographs were taken at 15 to 20 days after infection. They are missing for some host-construct combinations.

Supplementary Table 1 Total number of reads from the twelve RNA-seq libraries and mapping results to Phytophthora capsici reference genome.

Supplementary Table 2 Number of genes, with a GO-term attributed, or with a CRN or a RxLR annotation, in the whole genome of Phytophthora capsici, in the set of selected expressed genes and in the list of DEGs.

Supplementary Table 3 Primers of genes from Phytophthora capsici and Capsicum annuum used for RT-qPCR experiments and for Gateway cloning. Note: Gene accession numbers for P. capsici can be retrieved on https://mycocosm.jgi.doe.gov/Phyca11/Phyca11.home.html. CUST_2407, CUST_14997 and CUST_16519 are differentially expressed in RNA-seq. CUST_5402, CUST_15481 and CUST_17572 are not. Amplification efficiency of primer pairs was calculated based on the slope of the standard curve, using the equation: E (%) = (-1+10^(-1/slope))x100.

Supplementary Table 4 Pairwise comparison of Phytophthora capsici gene expression level between the fourhost-isolate interactions for the 7,240 selected genes according to the seven contrasts studied and their corresponding GO terms.

Supplementary Table 5 Number of up- and down-regulated Phytophthora capsici DEGs for each studied contrast (with FDR<0,01).

SupplementaryTable 6 Enriched and depleted GO-terms according to the seven considered contrasts, within the whole list of 709 DEGs.

Supplementary Table 7 Assignment of the 450 interaction-related DEGs to Cluster 1 and Cluster 2 of co-expressed Phytophthora capsici DEGs, and their corresponding GO terms. Note: (a) Genes assigned to cluster 0 do not belong to any cluster of co-expressed genes.

Supplementary Table 8 Enriched and depleted GO terms in Cluster1 and Cluster 2 of co-expressed Phytophthora capsici DEGs.

Supplementary Table 9 Number of points developing a hypersensitive response (HR) after PVX agro-infection with constructs CUST_2407 and CUST_16519 from isolates A and N, in leaves of seven resistant and four susceptible lines of pepper. Note: The table shows, for each host-construct combination, the number of PVX agro-infection points (obs columns), the number of infection points developing dark local necrosis (scored as a hypersensitive response or HR) (HR columns), and the percentage of infection points developing an HR (%_HR columns).The results of the EXP-1 and EXP-6 experiments are reported separately for ease of comparison. R and S (in the R.vs.S column) indicate pepper lines susceptible and lines partially resistant to Phytophthora capsici, respectively. The PVX column reports for each pepper line the mean relative concentration of PVX obtained in a PVX susceptibility assay; all lines were susceptible to PVX because their mean relative virus concentration was greater than three times the mean of non-inoculated controls (data not shown). The grey columns on the right add up for each pepper line the results for the sixagro-infected constructs. The grey lines sum the results of the resistant lines (Total_R), the susceptible lines (Total_S), the susceptible and resistant linesper experiment (Total S+R) and the both experiments (Total S + R Exp-1 + Exp-6). With constructs CUST_2407 and CUST_16519 from isolates A and N, host CM334 (R in the manuscript) showed yellowing followed by abscission of agro-infected leaves, which were assumed to correspond to an extreme resistance (ER). The pGR106::16240 and pGR106::GFP were used as positive and negative controls, respectively. Leaves of Nicotiona benthamiana did not show dark local necrosis to the six agro-infected constructs.

## Abbreviations

A, adapted isolate (Pc107); N, non-adapted isolate (Pc273); R, resistant host (CM334); S, susceptible host (Yolo Wonder); V8, mock inoculation with medium V8; [R-S], contrast of the two hosts on the *P. capsici* gene expression level irrespective of the isolate; [R_A-S_A], contrast of the two hosts on the isolate A gene expression level; [R_N-S_N], contrast of the two hosts on the isolate N gene expression level; [A-N], contrast of the two isolates on the *P. capsici* gene expression level irrespective of the host; [R_A-R_N], contrast of the two isolates on the *P. capsici* gene expression level in host R; [S_A-S_N], contrast of the two isolates on the *P. capsici* gene expression level in host S; [R_A-R_N]-[S_A-S_N], contrast of the two hosts crossed to the two isolates on the *P. capsici* gene expression level.

## References

[B1] Abrahamian M. Ah-Fong A. M. V. Davis C. Andreeva K. Judelson H. S. (2016). Gene expression and silencing studies in *Phytophthora infestans* reveal infection-specific nutrient transporters and a role for the nitrate reductase pathway in plant pathogenesis. PloS Pathog. 12 (12), e1006097. doi: 10.1371/journal.ppat.1006097 27936244 PMC5176271

[B2] Ah-Fong A. M. V. Kim K. S. Judelson H. S. (2017). RNA-Seq of life stages of the oomycete *Phytophthora infestans* reveals dynamic changes in metabolic, signal transduction, and pathogenesis genes and a major role for calcium signaling in development. BMC Genomics 18 (1), 198. doi: 10.1186/s12864-017-3585-x 28228125 PMC5322657

[B3] Bendahmane A. Kanyuka K. Baulcombe D. C. (1999). The rx gene from potato controls separate virus resistance and cell death responses. Plant Cell 11 (5), 781–791. doi: 10.1105/tpc.11.5.781 10330465 PMC144223

[B4] Bonnet J. Danan S. Boudet C. Barchi L. Sage-Palloix A.-M. Caromel B.. (2007). Are the polygenic architectures of resistance to *Phytophthora capsici* and p. parasitica independent in pepper? Theor. Appl. Genet. 115 (2), 253–264. doi: 10.1007/s00122-007-0561-x 17497121

[B5] Castro-Rocha A. Shrestha S. Lyon B. Grimaldo-Pantoja G. L. Flores-Marges J. P. Valero-Galván J.. (2016). An initial assessment of genetic diversity for *Phytophthora capsici* in northern and central Mexico. Mycol Prog. 15 (2), 15. doi: 10.1007/s11557-016-1157-0

[B6] Chen X. R. Xing Y. P. Li Y. P. Tong Y. H. Xu J. Y. (2013). RNA-Seq reveals infection-related gene expression changes in *Phytophthora capsici* . PloS One 8 (9), e74588. doi: 10.1371/journal.pone.0074588 24019970 PMC3760852

[B7] Cowger C. Brown J. K. M. (2019). Durability of quantitative resistance in crops: Greater than we know? Annu. Rev. Phytopathol. 57 (1), 253–277. doi: 10.1146/annurev-phyto-082718-100016 31206351

[B8] Delmotte F. Mestre P. Schneider C. Kassemeyer H. H. Kozma P. Richart-Cervera S.. (2014). Rapid and multiregional adaptation to host partial resistance in a plant pathogenic oomycete: Evidence from European populations of *Plasmopara viticola*, the causal agent of grapevine downy mildew. Infect Genet. Evol. 27, 500–508. doi: 10.1016/j.meegid.2013.10.017 24184095

[B9] Dixon R. (2001). Natural products and plant disease resistance. Nature 411 (6839), 843–847. doi: 10.1038/35081178 11459067

[B10] Dobin A. Davis C. A. Schlesinger F. Drenkow J. Zaleski C. Jha S.. (2013). STAR: Ultrafast universal RNA-seq aligner. Bioinformatics 29 (1), 15–21. doi: 10.1093/bioinformatics/bts635 23104886 PMC3530905

[B11] Du J. Rietman H. Vleeshouwers V. G. A. A. (2014). Agroinfiltration and PVX agroinfection in potato and *Nicotiana benthamiana* . J. Visualized Experiment 83 (e50971), 1–7. doi: 10.3791/50971 PMC4063549 24430891

[B12] Edgar R. Domrachev M. Lash A. E. (2002). Gene expression omnibus: NCBI gene expression and hybridization array data repository. Nucleic Acids Res. 30 (1), 207–210. doi: 10.1093/nar/30.1.207 11752295 PMC99122

[B13] Fawke S. Doumane M. Schornack S. (2015). Oomycete interactions with plants: Infection strategies and resistance principles. Microbiol. Mol. Biol. Rev. 79 (3), 263–280. doi: 10.1128/mmbr.00010-15 26041933 PMC4468149

[B14] Gagnot S. Tamby J. P. Martin-Magniette M. L. Bitton F. Taconnat L. Balzergue S.. (2008). CATdb: A public access to arabidopsis transcriptome data from the URGV-CATMA platform. Nucleic Acids Res. 36 (SUPPL. 1), 986–990. doi: 10.1093/nar/gkm757 PMC2238931 17940091

[B15] Haas B. J. Kamoun S. Zody M. C. Jiang R. H. Y. Handsaker R. E. Cano L. M.. (2009). Genome sequence and analysis of the Irish potato famine pathogen *Phytophthora infestans* . Nature 461 (7262), 393–398. doi: 10.1038/nature08358 19741609

[B16] Hématy K. Cherk C. Somerville S. (2009). Host–pathogen warfare at the plant cell wall. Curr. Opin. Plant Biol. 12 (4), 406–413. doi: 10.1016/j.pbi.2009.06.007 19616468

[B17] Jupe J. Stam R. Howden A. J. M. Morris J. Zhang R. Hedley P. E.. (2013). *Phytophthora capsici*-tomato interaction features dramatic shifts in gene expression associated with a hemi-biotrophic lifestyle. Genome Biol. 14 (6), R63. doi: 10.1186/gb-2013-14-6-r63 23799990 PMC4054836

[B18] Kamoun S. (2006). A catalogue of the effector secretome of plant pathogenic oomycetes. Annu. Rev. Phytopathol. 44 (1), 41–60. doi: 10.1146/annurev.phyto.44.070505.143436 16448329

[B19] Kellner R. Bhattacharyya A. Poppe S. Hsu T. Y. Brem R. B. Stukenbrock E. H. (2014). Expression profiling of the wheat pathogen *Zymoseptoria tritici* reveals genomic patterns of transcription and host-specific regulatory programs. Genome Biol. Evol. 6 (6), 1353–1365. doi: 10.1093/gbe/evu101 24920004 PMC4079195

[B20] Kim S. Park J. Yeom S. I. Kim Y. M. Seo E. Kim K. T.. (2017). New reference genome sequences of hot pepper reveal the massive evolution of plant disease-resistance genes by retroduplication. Genome Biol. 18 (1), 1–11. doi: 10.1186/s13059-017-1341-9 29089032 PMC5664825

[B21] Kopylova E. Noé L. Touzet H. (2012). SortMeRNA: Fast and accurate filtering of ribosomal RNAs in metatranscriptomic data. Bioinformatics 28 (24), 3211–3217. doi: 10.1093/bioinformatics/bts611 23071270

[B22] Kushalappa A. C. Yogendra K. N. Karre S. (2016). Plant innate immune response: Qualitative and quantitative resistance. Crit. Rev. Plant Sci. 35 (1), 38–55. doi: 10.1080/07352689.2016.1148980

[B23] Laluk K. Mengiste T. (2010). Necrotroph attacks on plants: Wanton destruction or covert extortion?The arabidopsis book 8, e01036. doi: 10.1199/tab.0136 PMC3244965 22303261

[B24] Lambert I. Paysant-Le Roux C. Colella S. Martin-Magniette M. L. (2020). DiCoExpress: A tool to process multifactorial RNAseq experiments from quality controls to co-expression analysis through differential analysis based on contrasts inside GLM models. Plant Methods 16 (1), 1–10. doi: 10.1186/s13007-020-00611-7 32426025 PMC7216733

[B25] Lamour K. H. Mudge J. Gobena D. Hurtado-gonzales O. P. Schmutz J. Kuo A.. (2012). Genome sequencing and mapping reveal loss of heterozygosity as a mechanism for rapid adaptation in the vegetable pathogen *Phytophthora capsici* . Mol. Plant-Microbe Interactions : MPMI 25 (10), 1350–1360. doi: 10.1094/MPMI-02-12-0028-R 22712506 PMC3551261

[B26] Lee Y. H. Kim H. S. Kim J. Y. Jung M. Park Y. S. Lee J. S.. (2004). A new selection method for pepper transformation: Callus-mediated shoot formation. Plant Cell Rep. 23 (1–2), 50–58. doi: 10.1007/s00299-004-0791-1 15221276

[B27] Lee J.-H. Siddique M. I. Kwon J.-K. Kang B.-C. (2021). Comparative genomic analysis reveals genetic variation and adaptive evolution in the pathogenicity-related genes of *Phytophthora capsici* . Front. Microbiol. 12. doi: 10.3389/fmicb.2021.694136 PMC8415033 34484141

[B28] Lefebvre V. Palloix A. (1996). Both epistatic and additive effects of QTLs are involved in polygenic induced resistance to disease: A case study , the interaction pepper - *Phytophthora capsici* leonian. Theor. Appl. Genet. 93 (4), 503–511. doi: 10.1007/s001220050308 24162341

[B29] Livak K. J. Schmittgen T. D. (2001). Analysis of relative gene expression data using real-time quantitative PCR and the 2-ΔΔCT method. Methods 25 (4), 402–408. doi: 10.1006/meth.2001.1262 11846609

[B30] Loisel E. Goncalves I. Poussereau N. Grosjean-Cournoyer M.-C. Villalba F. Bruel C. (2016). Transcriptional study of the ABC transporter-encoding genes in response to fungicide treatment and during plant infection in the phytopathogenic fungus botrytis cinerea. BioRxiv, 095869. doi: 10.1101/095869

[B31] Maculins T. Fiskin E. Bhogaraju S. Dikic I. (2016). Bacteria-host relationship: Ubiquitin ligases as weapons of invasion. Cell Res. 26 (4), 499–510. doi: 10.1038/cr.2016.30 26964724 PMC4822128

[B32] Magori S. Citovsky V. (2011). Hijacking of the host SCF ubiquitin ligase machinery by plant pathogens. Front. Plant Sci. 2 (NOV). doi: 10.3389/fpls.2011.00087 PMC3355745 22645554

[B33] Malinovsky F. G. Fangel J. U. Willats W. G. T. (2014). The role of the cell wall in plant immunity. Front. Plant Sci. 5 (May). doi: 10.3389/fpls.2014.00178 PMC4018530 24834069

[B34] McCarthy D. J. Chen Y. Smyth G. K. (2012). Differential expression analysis of multifactor RNA-seq experiments with respect to biological variation. Nucleic Acids Res. 40 (10), 4288–4297. doi: 10.1093/nar/gks042 22287627 PMC3378882

[B35] McDonald B. A. Linde C. (2002). Pathogen population genetics, evolutionary potential, and durable resistance. Annu. Rev. Phytopathol. 40 (1), 349–379. doi: 10.1146/annurev.phyto.40.120501.101443 12147764

[B36] Meijer H. J. G. Hassen H. H. Govers F. (2011). *Phytophthora infestans* has a plethora of phospholipase d enzymes including a subclass that has extracellular activity. PloS One 6 (3), 1–12. doi: 10.1371/journal.pone.0017767 PMC3056787 21423760

[B37] Muszewska A. Stepniewska-Dziubinska M. M. Steczkiewicz K. Pawlowska J. Dziedzic A. Ginalski K. (2017). Fungal lifestyle reflected in serine protease repertoire. Sci. Rep. 7 (1), 1–12. doi: 10.1038/s41598-017-09644-w 28831173 PMC5567314

[B38] Olsson V. Butenko M. A. (2018). Abscission in plants. Curr. Biol. 28 (8), R338–R339. doi: 10.1016/j.cub.2018.02.069 29689203

[B39] Palloix A. Ayme V. Moury B. (2009). Durability of plant major resistance genes to pathogens depends on the genetic background, experimental evidence and consequences for breeding strategies. New Phytol. 183 (1), 190–199. doi: 10.1111/j.1469-8137.2009.02827.x 19344475

[B40] Pang Z. Chen L. Mu W. Liu L. Liu X. (2016). Insights into the adaptive response of the plant-pathogenic oomycete *Phytophthora capsici* to the fungicide flumorph. Sci. Rep. 6 (April), 1–9. doi: 10.1038/srep24103 27050922 PMC4822174

[B41] Patharkar O. R. Gassmann W. Walker J. C. (2017). Leaf shedding as an anti-bacterial defense in arabidopsis cauline leaves. PloS Genet. 13 (12), 1–18. doi: 10.1371/journal.pgen.1007132 PMC5749873 29253890

[B42] Reyes-Tena A. Huguet-Tapia J. C. Lamour K. H. Goss E. M. Rodríguez-Alvarado G. Vázquez-Marrufo G.. (2019). Genome sequence data of six isolates of *Phytophthora capsici* from Mexico. Mol. Plant-Microbe Interactions® 32 (10), 1267–1269. doi: 10.1094/MPMI-01-19-0014-A 31425006

[B43] Robinson M. D. McCarthy D. J. Smyth G. K. (2010). edgeR: a bioconductor package for differential expression analysis of digital gene expression data. Bioinformatics 26 (1), 139–140. doi: 10.1093/bioinformatics/btp616 19910308 PMC2796818

[B44] Schornack S. Huitema E. Cano L. M. Bozkurt T. O. Oliva R. Van Damme M.. (2009). Ten things to know about oomycete effectors. Mol. Plant Pathol. 10 (6), 795–803. doi: 10.1111/j.1364-3703.2009.00593.x 19849785 PMC6640533

[B45] Singer A. U. Schulze S. Skarina T. Xu X. Cui H. Eschen-Lippold L.. (2013). A pathogen type III effector with a novel E3 ubiquitin ligase architecture. PloS Pathog. 9 (1), e1003121. doi: 10.1371/journal.ppat.1003121 23359647 PMC3554608

[B46] Sprockett D. D. Piontkivska H. Blackwood C. B. (2011). Evolutionary analysis of glycosyl hydrolase family 28 (GH28) suggests lineage-specific expansions in necrotrophic fungal pathogens. Gene 479 (1–2), 29–36. doi: 10.1016/j.gene.2011.02.009 21354463

[B47] Stam R. Jupe J. Howden A. J. M. Morris J. A. Boevink P. C. Hedley P. E.. (2013). Identification and characterisation CRN effectors in *Phytophthora capsici* shows modularity and functional diversity. PloS One 8 (3), 1–13. doi: 10.1371/journal.pone.0059517 PMC3607596 23536880

[B48] Stefańczyk E. Sobkowiak S. Brylińska M. Śliwka J. (2017). Expression of the potato late blight resistance gene *Rpi-phu1* and *Phytophthora infestans* effectors in the compatible and incompatible interactions in potato. Phytopathology 107 (6), 740–748. doi: 10.1094/PHYTO-09-16-0328-R 28134594

[B49] Thabuis A. Palloix A. Pflieger S. Daubèze A. M. Caranta C. Lefebvre V. (2003). Comparative mapping of *Phytophthora* resistance loci in pepper germplasm: evidence for conserved resistance loci across solanaceae and for a large genetic diversity. TAG. Theor. Appl. Genet. 106 (8), 1473–1485. doi: 10.1007/S00122-003-1206-3 12750791

[B50] Tripodi P. Rabanus-Wallace M. T. Barchi L. Kale S. Esposito S. Acquadro A.. (2021). Global range expansion history of pepper (*Capsicum* spp.) revealed by over 10,000 genebank accessions. Proc. Natl. Acad. Sci. United States America 118 (34), e21043315118. doi: 10.1073/pnas.2104315118 PMC8403938 34400501

[B51] Vleeshouwers V. G. A. A. Raffaele S. Vossen J. H. Champouret N. Oliva R. Segretin M. E.. (2011). Understanding and exploiting late blight resistance in the age of effectors. Annu. Rev. Phytopathol. 49 (May), 507–531. doi: 10.1146/annurev-phyto-072910-095326 21663437

[B52] Wang S. Boevink P. C. Welsh L. Zhang R. Whisson S. C. Birch P. R. J. (2017). Delivery of cytoplasmic and apoplastic effectors from *Phytophthora infestans* haustoria by distinct secretion pathways. New Phytol. 216 (1), 205–215. doi: 10.1111/nph.14696 28758684 PMC5601276

[B53] Yan H. Z. Liou R. F. (2006). Selection of internal control genes for real-time quantitative RT-PCR assays in the oomycete plant pathogen *Phytophthora parasitica* . Fungal Genet. Biol. 43 (6), 430–438. doi: 10.1016/j.fgb.2006.01.010 16531084

[B54] Zuluaga A. P. Vega-Arreguín J. C. Fei Z. Matas A. J. Patev S. Fry W. E.. (2016a). Analysis of the tomato leaf transcriptome during successive hemibiotrophic stages of a compatible interaction with the oomycete pathogen *Phytophthora infestans* . Mol. Plant Pathol. 17 (1), 42–54. doi: 10.1111/mpp.12260 25808779 PMC6638369

[B55] Zuluaga A. P. Vega-Arreguín J. C. Fei Z. Ponnala L. Lee S. J. Matas A. J.. (2016b). Transcriptional dynamics of *Phytophthora infestans* during sequential stages of hemibiotrophic infection of tomato. Mol. Plant Pathol. 17 (1), 29–41. doi: 10.1111/mpp.12263 25845484 PMC6638332

