# Supplementary figures and images for: Strive or thrive: Trends in Phytophthora capsici gene expression in partially resistant pepper
Source: Front Plant Sci. 2022 Nov 21;13:980587. doi: 10.3389/fpls.2022.980587 (PMC9721114; doi:10.3389/fpls.2022.980587)

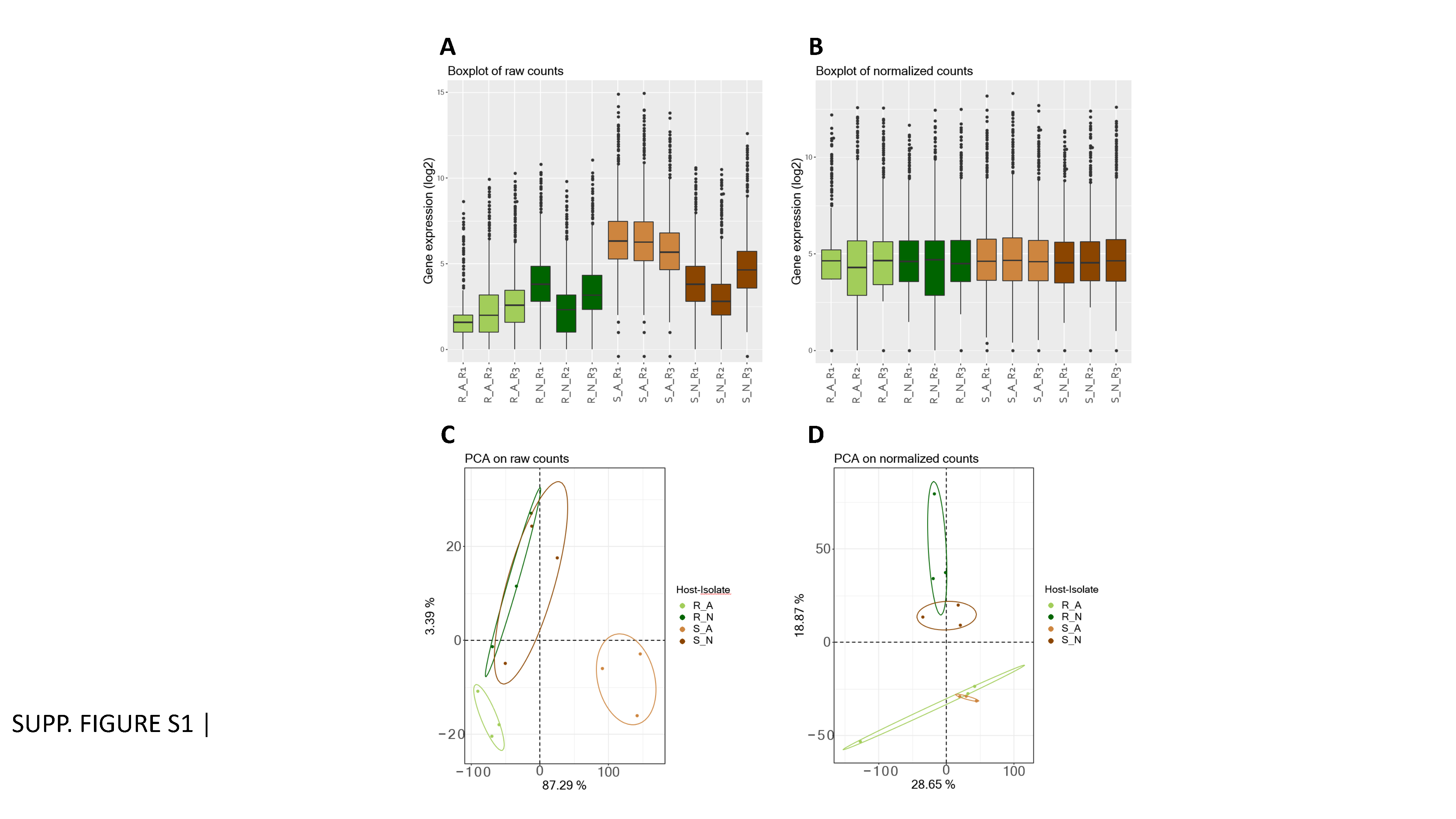

Supplement: Supplementary Figure 1 — Data quality control of the twelve sample libraries. Boxplot of the library sizes for each sample (A) before and (B) after the TMM-normalization, respectively. First and second axes of the Principal Component Analysis (PCA) (C) on the raw and (D) normalized counts. A, adapted isolate (Pc107); N, non-adapted isolate (Pc273); R, resistant host (CM334); S, susceptible host (Yolo Wonder). R1, R2 and R3 are samples from the three independent replicates. On PCAs, the three dots of a same color correspond to three independent replicates for a specific host-isolate interaction. [file Image_1.tiff]

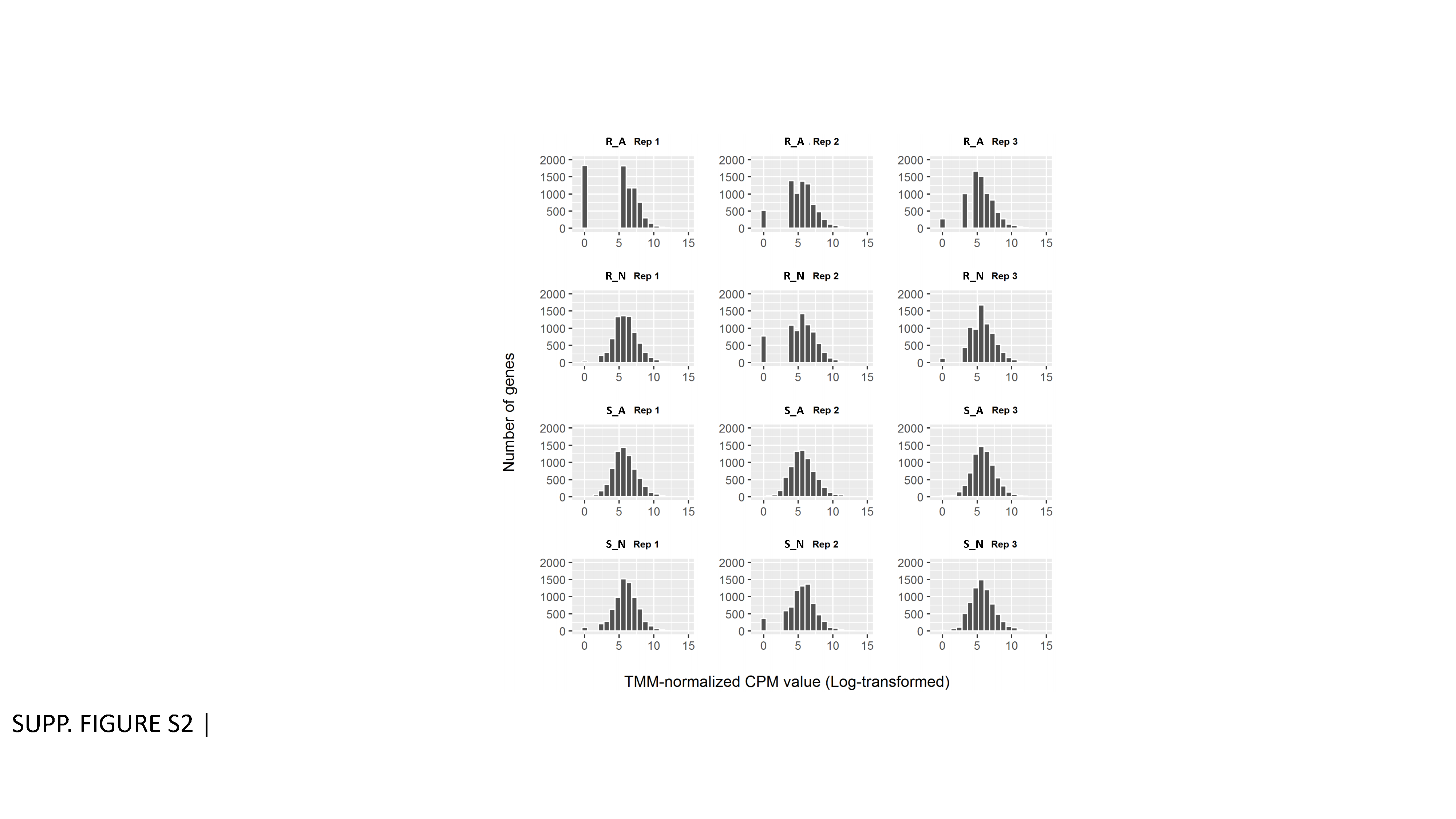

Supplement: Supplementary Figure 2 — Gene distribution by sample according to their expression level. The level of expression for a gene is expressed by the log-transformation of its TMM-normalized count per million values (TMM-CPM). The three biological replicates (Rep) are represented in column. The four host-isolate interactions are represented in line. A, adapted isolate (Pc107); N, non-adapted isolate (Pc273); R, resistant host (CM334); S, susceptible host (Yolo Wonder). [file Image_2.tiff]

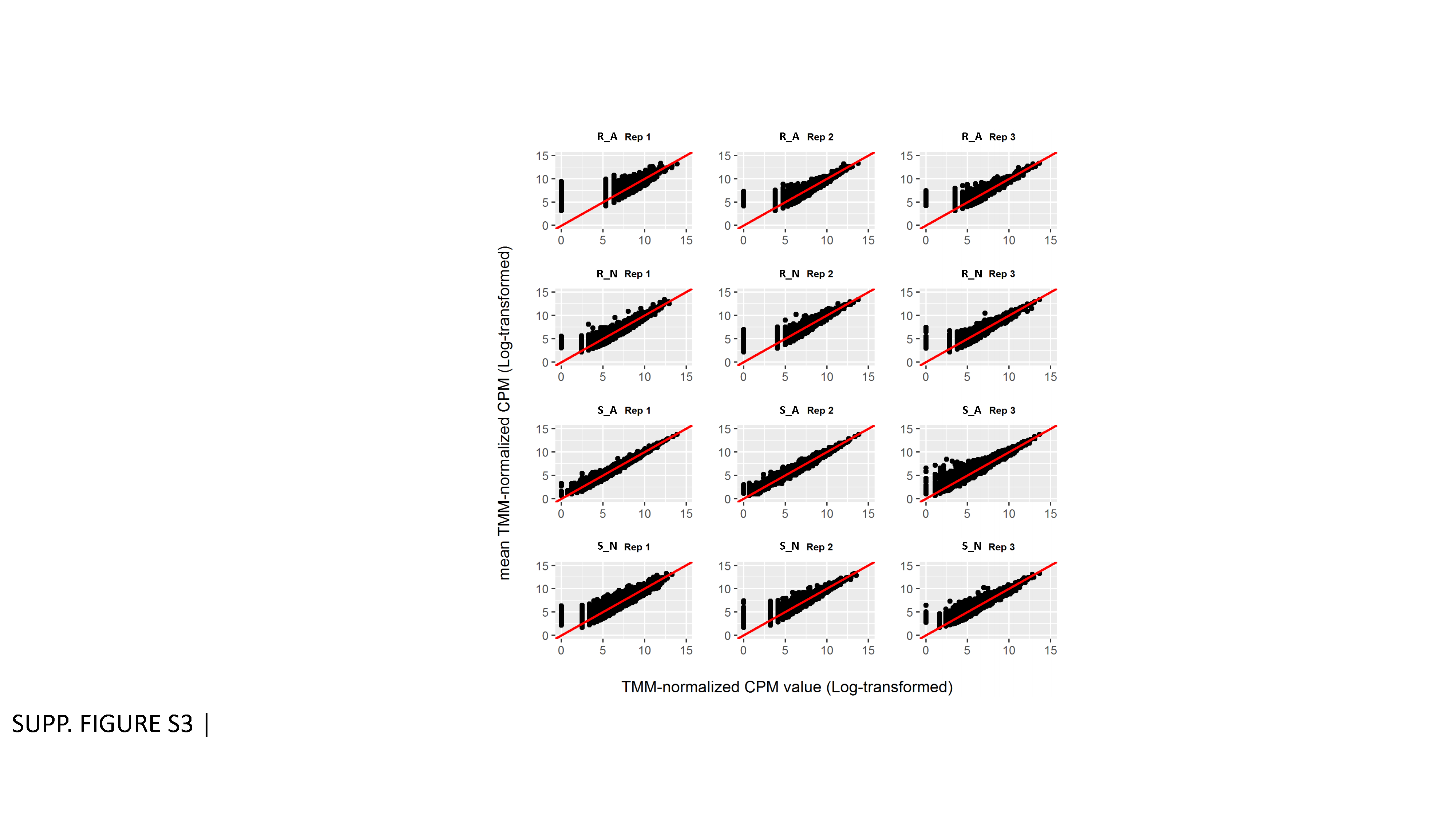

Supplement: Supplementary Figure 3 — Projection of the mean gene expression of a host-isolate-interaction (y-axis) on the gene expression of a single replicate from the same host-isolate interaction (x-axis). The level of gene expression is expressed by the log-transformation of its TMM-normalized count per million values (TMM-CPM). The projections of the three biological replicates (Rep) are represented in column. The four host-isolate interactions are represented in line. A, adapted isolate (Pc107); N, non-adapted isolate (Pc273); R, resistant host (CM334); S, susceptible host (Yolo Wonder). [file Image_3.tiff]

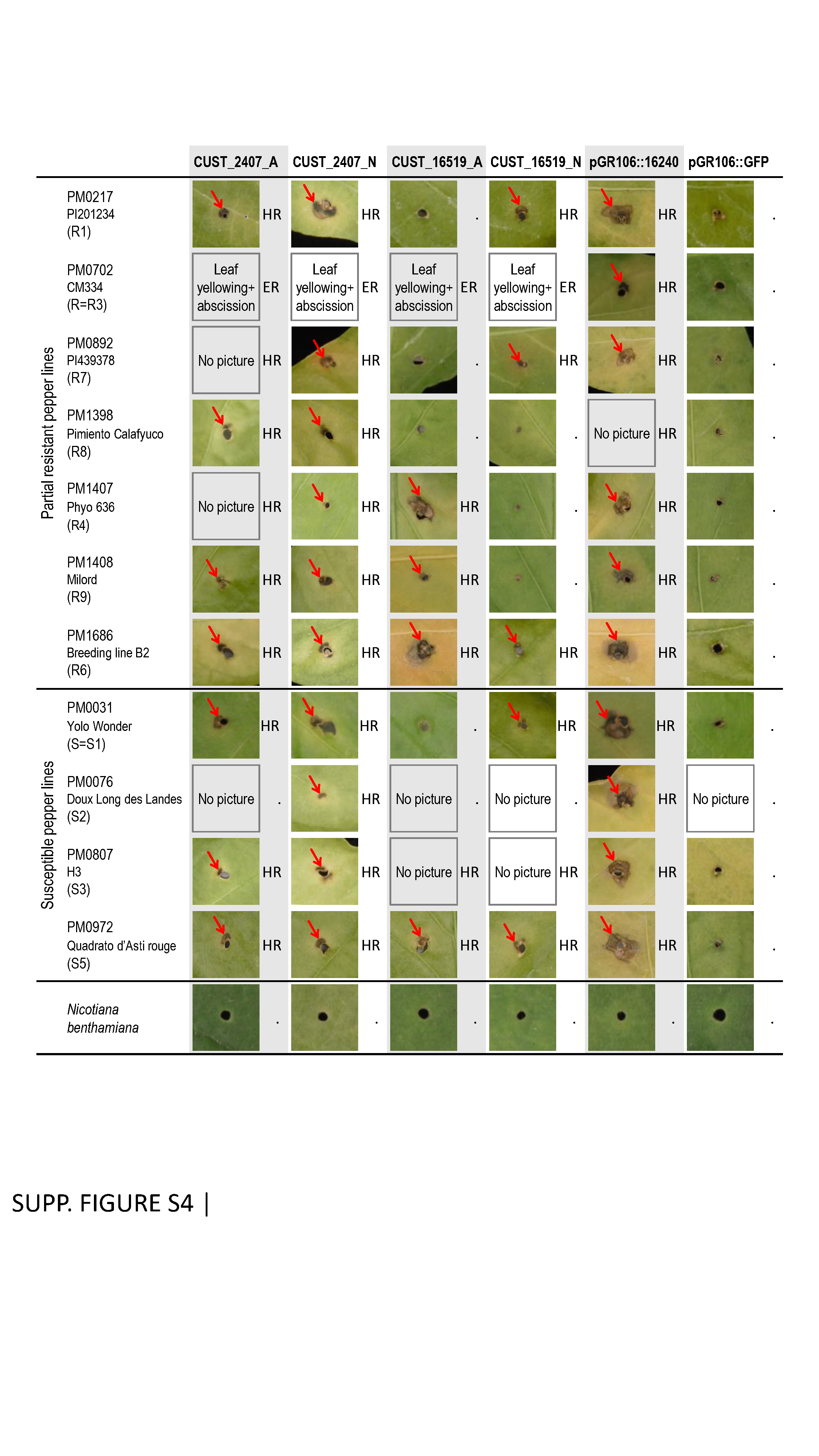

Supplement: Supplementary Figure 4 — Symptoms of transient in planta expression of RxLR genes CUST_2407 and CUST_16519 from isolates A and N in leaves of seven resistant and four susceptible pepper lines after PVX agro-infection. Red arrows indicate the dark local necrosis observed around the agro-infection point, scored as a hypersensitive response (HR). For each host-RxLR combination, HR on the right side of the photograph means that a HR was observed around at least one infection point, in either the Exp-1 or the Exp-6 experiment. A dot means that no HR was observed. With constructs CUST_2407 and CUST_16519 from isolates A and N, host CM334 (R in the manuscript) showed yellowing followed by abscission of agro-infected leaves, which were assumed to correspond to extreme resistance (ER). The constructs pGR106::16240 and pGR106::GFP were used as positive and negative controls, respectively. Leaves of Nicotiana benthamiana did not show dark local necrosis with the six agro-infected constructs. Photographs were taken at 15 to 20 days after infection. They are missing for some host-construct combinations. [file Image_4.tiff]
